# A Novel R2R3-MYB Gene *LoMYB33* From Lily Is Specifically Expressed in Anthers and Plays a Role in Pollen Development

**DOI:** 10.3389/fpls.2021.730007

**Published:** 2021-09-23

**Authors:** Xinyue Liu, Ze Wu, Jingxian Feng, Guozhen Yuan, Ling He, Dehua Zhang, Nianjun Teng

**Affiliations:** ^1^Key Laboratory of Landscaping Agriculture, Ministry of Agriculture and Rural Affairs, College of Horticulture, Nanjing Agricultural University, Nanjing, China; ^2^Key Laboratory of Biology of Ornamental Plants in East China, National Forestry and Grassland Administration, College of Horticulture, Nanjing Agricultural University, Nanjing, China; ^3^College of Agriculture, Nanjing Agricultural University, Nanjing, China

**Keywords:** lily, R2R3-MYB, *LoMYB33*, pollen, anther development

## Abstract

Lily (*Lilium* spp.) is an important commercial flower crop, but its market popularity and applications are adversely affected by severe pollen pollution. Many studies have examined pollen development in model plants, but few studies have been conducted on flower crops such as lily. GAMYBs are a class of R2R3-MYB transcription factors and play important roles in plant development and biotic resistance; their functions vary in different pathways, and many of them are involved in anther development. However, their function and regulatory role in lily remain unclear. Here, the *GAMYB* homolog *LoMYB33* was isolated and identified from lily. The open reading frame of *LoMYB33* was 1620 bp and encoded a protein with 539 amino acids localized in the nucleus and cytoplasm. Protein sequence alignment showed that LoMYB33 contained a conserved R2R3 domain and three BOX motifs (BOX1, BOX2, and BOX3), which were unique to the GAMYB family. LoMYB33 had transcriptional activation activity, and its transactivation domain was located within 90 amino acids of the C-terminal. *LoMYB33* was highly expressed during the late stages of anther development, especially in pollen. Analysis of the promoter activity of *LoMYB33* in transgenic Arabidopsis revealed that the *LoMYB33* promoter was highly activated in the pollen of stage 12 to 13 flowers. Overexpression of *LoMYB33* in Arabidopsis significantly retarded growth; the excess accumulation of *LoMYB33* also negatively affected normal anther development, which generated fewer pollen grains and resulted in partial male sterility in transgenic plants. Silencing of *LoMYB33* in lily also greatly decreased the amount of pollen. Overall, our results suggested that *LoMYB33* might play an important role in the anther development and pollen formation of lily.

## Introduction

Pollen is required for sexual reproduction in plants (Aizen and Harder, 2007). Normal pollen development is essential for ensuring the high yield and quality of crops and fruits, but deleterious effects are often inevitable. For example, many ornamental plants (e.g., *Populus*, lily, and chrysanthemum) contain high concentrations of pollen, which results in pollen contamination and anaphylactic reactions in susceptible populations and reduces the commercial value of these ornamental plants (Tong et al., 2013; Sui et al., 2015; Wang et al., 2016; Kim et al., 2018; Feng et al., 2019; Li et al., 2019; Teng et al., 2021). Thus, male-sterile or pollen-free ornamental plants are often highly useful. Although many studies have examined pollen development in model plants (Köhler et al., 2003; Millar and Gubler, 2005; Chu, 2006; Long et al., 2008), studies of flower crops are relatively scarce.

Gibberellins (GAs) are a major group of hormones in plants that play an essential role in plant vegetative growth and reproductive development (Hedden, 2001). GAMYBs are members of the R2R3-MYB family; in addition to the conserved R2R3 domain at the N-terminus (Woodger et al., 2003), the GAMYB transcription factors (TFs) contain three BOX motifs, an important feature of this family (Dubos et al., 2010). In 1995, GAMYBs were first reported in barley aleurone cells, and they were shown to be up-regulated by gibberellin (GA) and activated GA-regulated genes (Gubler et al., 1995). GAMYBs also play important roles in flower induction and flowering regulation. For example, AtMYB33 in Arabidopsis directly binds to the promoter of *AtLFY* to regulate flowering (Gocal et al., 2001); CsGAMYB affects the differentiation of male and female flowers in cucumber (Zhang et al., 2014). HvGAMYB also plays a role in the formation of male organs in barley (Murray et al., 2003). In addition, GAMYBs have been reported to regulate anther development in a variety of plants (Kaneko et al., 2004). Both AtMYB33 and AtMYB65 are GAMYB family members in Arabidopsis, which function redundantly to regulate anther development and pollen formation (Millar and Gubler, 2005). Rice *gamyb* mutants are sterile because the anthers are poorly developed and lack pollen (Aya et al., 2009).

In Arabidopsis, *AtMYB33* and *AtMYB65* are targeted by miRNA159; as the silencing or overexpression of miRNA159 can interfere with anther development, miRNA159 has been suggested to be a homeostatic modulator of GAMYB activity ensuring normal anther development (Achard et al., 2004). In rice, GA activates OsGAMYB in the tapetum through the GID1/DELLA sensing pathway, which leads to the up-regulation of *OsCYP703A* and *OsKAR* for sporopollen formation (Cheng et al., 2004; Kwon et al., 2015). Sporopollen is the key component of the pollen outer wall (Dickinson and Heslop-Harrison, 1968), and its absence causes pollen abortion. These results indicate that GAMYBs are required for normal pollen development in model plants, but their roles in ornamental plants remain unknown.

Lily (*Lilium* spp.) is an important commercial flower crop (Gong et al., 2014), but its market popularity and applications are adversely affected by severe pollen pollution. There is thus a need to explore the mechanism of pollen development in lilies with little or no pollen (Wang et al., 2019; Yuan et al., 2021). According to our previous transcriptome data of pollen abortion varieties (Wang et al., 2019), an *MYB* gene annotated as GAMYB (*LoMYB33*) is differentially expressed in two periods before and after pollen abortion, indicating that *LoMYB33* may be involved in pollen development. Here, a GAMYB member in lily (LoMYB33) was isolated and identified. LoMYB33 showed transactivation ability, and it located in the cytoplasm and nucleus; its C-terminal contributed to its transactivation ability. *LoMYB33* was highly expressed in the late stages of anther development, especially in pollen. *LoMYB33* overexpression in Arabidopsis resulted in significant growth retardation and reduced the number of normal pollen grains, which caused male sterility in transgenic plants. The silencing of *LoMYB33* in lily also reduced the number of pollen. These results indicate that LoMYB33 plays a role in pollen development; its function thus requires strict control under normal development conditions.

## Materials and Methods

### Plant Materials and Growth Conditions

The *Lilium* Oriental hybrid “Siberia” and *Lilium longiflorum* “White Heaven” were used as the experimental materials. “Siberia” was planted in the Baguazhou Lily Germplasm Resource Base of Nanjing Agricultural University and planted in a greenhouse with sufficient sunlight at ~20°C. “White Heaven” was grown in a growth room at 22°C with a 16 h:8 h light:dark photoperiod. Both Arabidopsis (Col-0) and tobacco (*Nicotiana benthamiana*) were used as the testing platform. Arabidopsis and tobacco seeds were sterilized with 1% sodium hypochlorite for 15 min, washed with sterile water 5–6 times, and sowed on MS medium (Murashige and Skoog, 1962). The seeds were placed in the dark at 4°C for 3 days and then transferred to a light incubator at 22°C (16 h/8 h light period). After 10 days, seedlings with four true leaves were transferred into a pot filled with peat and vermiculite (1:1) and then cultured in a controlled environment as described above in subsequent experiments.

### Isolation of *LoMYB33* From Lily

Anthers in the 12-cm length flower buds were collected, and total RNA was extracted with an RNAprep Pure Plant Kit (DP-103, Tiangen, China). cDNA was synthesized by M-MLV reverse transcriptase (R233-01, Vazyme, China) with oligo-dT primers. Based on transcriptome data (Wang et al., 2019), the specific primers for the *LoMYB33* coding sequence (Supplementary Table 1) were designed, and the fragment was isolated using PrimeSTAR HS DNA polymerase (DR010A, Takara, Japan). The target fragment was constructed into the pMD18-T vector and sequenced.

### Multiple Sequence Alignment and Phylogenetic Analysis

The phylogenetic tree of different species, the phylogenetic tree of the MYB family of Arabidopsis, and the phylogenetic tree of LoMYB33 and some MYB proteins in rice were constructed using the neighbor-joining method in MEGA7 (Kumar et al., 2016). The sequences of Arabidopsis MYB members were downloaded from the TAIR database (https://www.arabidopsis.org/index.jsp). The sequences of rice MYB members were downloaded from the NCBI database (https://www.ncbi.nlm.nih.gov/). The protein multiple alignment of LoMYB33 with other GAMYB members from different species was conducted using ClustalX 1.81 and BioEdit 7.0 software (Hall, 1999; Thompson et al., 2003).

### Transcription Activation Activity Analysis

The yeast system was used for transactivation analysis. The recombinant plasmids, the positive control GAL4, and the negative control pGBKT7 were transformed into yeast AH109 cells and spread on SD/-Trp medium. The transformed yeast cells were incubated at 30°C for 3 days. The transcriptional activity was evaluated by growing yeast colonies on SD/-Trp/-His and SD/-Trp/-His with 5 mM 3-AT (3-amino-1,2,4-triazole)-deficient solid media. The transformed yeast cells grown on SD/-Trp medium were also transferred to filter paper for transcriptional activity analysis, and X-α-Gal was added to observe the activity of β-galactosidase (Ding et al., 2021).

### Subcellular Localization Analysis

*LoMYB33* or *mLoMYB33* (with mutated target sites of miRNA159) was cloned into the pCAMBIA1300-green fluorescent protein (GFP) vector. The recombinant plasmid was then transformed into *Agrobacterium tumefaciens* strain GV3101. Five μL of *Agrobacterium* competent cells harboring plasmids was mixed gently, placed on ice for 5 min, and rapidly frozen for 1 min in liquid nitrogen, followed by treatment with 37°C for 5 min and cooled on ice for 2 min. The cells were then cultured with LB medium at 28°C and 200 rpm for 3–5 h. After centrifugation and resuspending the bacteria, they were evenly spread on solid LB medium (containing 50 μg·μL^−1^ Kan and 100 μg·μL^−1^ Rif) and cultured at 28°C for 2 days. The appropriate clones were then selected and cultured with liquid LB medium (containing 50 μg·μL^−1^ Kan and 100 μg·μL^−1^ Rif) for 12 h. The bacterial solution was resuspended with the injection buffer (10 mM MgCl_2_, 100 mM 2-morpholinoethanesulfonic acid, and 200 μM acetosyringone, pH = 5.8) and injected into tobacco leaves (Wu et al., 2019). After 48 h, the GFP signal was observed with a laser scanning confocal microscope (LSM800, Zeiss, Germany).

### Gene Expression Analysis

Total RNA was extracted from the bulbus, root, stem, leaf, petal, and anthers at different developmental stages, as well as the ovary of lily using the Trizol method (TaKaRa, Japan). Reverse transcription was performed using a HiScript II kit (R233-01, Vazyme, China). Real-time quantitative PCR (RT-qPCR) was used to determine the expression levels. The *18S* rRNA of lily was used as the reference gene. The primers are shown in Supplementary Table 1. The SYBR^®^ Green Realtime PCR reaction system was used for RT-qPCR. The cycling parameters were as follows: 95°C for 2 min; 95°C for 15 s, 55°C for 15 s, and 72°C for 20 s for 40 cycles. The CT value of each sample was obtained, and quantitative analysis of the relative level of expression was performed using the 2^−ΔΔCt^ method (Livak and Schmittgen, 2001).

### GA_3_ Treatment

Lily “Siberia” plants at the same development stage were treated with 288 μM GA_3_ in a bottle; a water treatment group subjected to the same conditions was used as a control. Samples were taken at 0, 4, 8, 12, 24, and 48 h after treatment. Three anthers with bud lengths <5 cm were sampled for RT-qPCR analysis.

### Isolation and Analysis of the *LoMYB33* Promoter

The *LoMYB33* promoter was cloned using the hiTAIL-PCR method (Liu and Chen, 2007) with three special reverse primers at the 5′ end of the *LoMYB33* and the five universal primers LAD1–5 and AC1 (Supplementary Table 1). The upstream fragment located 465 bp away from the ATG of *LoMYB33* was isolated and identified. The promoter sequence was analyzed using New PLACE software (https://www.dna.affrc.go.jp/PLACE/?action=newplace).

### Analysis of Promoter Activity With the GUS Reporter

The promoter fragment (465 bp) of *LoMYB33* was inserted into the pCAMBIA1391 vector. The constructed vector was transformed into *A. tumefaciens* strain GV3101. The floral-dip method was used for Arabidopsis transformation (Clough and Bent, 1998). The *proLoMYB33*:GUS transgenic plants were screened on MS medium containing 35 mg L^−1^ hygromycin. For histochemical GUS analysis, plant tissues were cultured in GUS staining solution (Huayueyang, Beijing, China) overnight at 37°C. Chlorophyll was then removed with 70% ethanol for imaging.

### Stable Transformation of Arabidopsis

The open reading frame (ORF) of *LoMYB33* was cloned and inserted into the pCAMBIA1300 vector and transformed into GV3101. Five-week-old Arabidopsis plants were used for transformation by the floral-dip method. The transgenic lines were identified by RT-PCR; three T3-generation homozygous lines were selected for the functional analysis. The primers used for the transgene identification are listed in Supplementary Table 1.

### Paraffin Sectioning and Histological Observation

The flower buds and anthers of wild-type and *LoMYB33*-overexpressing Arabidopsis plants at different developmental stages were vacuum-infiltrated with FAA solution. Fixed anthers were dehydrated in 50, 70, 85, 90, and 100% ethanol gradients (2 h each) and then embedded in paraffin. Paraffin sections (8 μm thick) were obtained with a microtome. The sections were stained with hematoxylin and observed using a light microscope (DM-6B, Leica, Germany).

### Characterization of Plant Phenotypes

Flowers were photographed under a stereomicroscope (M165FC, Leica, Germany). To determine pollen viability, anthers at stage 12 (Sanders et al., 1999) were collected and stained with Alexander solution (Alexander, 1969) and observed under an optical electron microscope (DM-6B, Leica, Germany).

### Determination of the Amount of Pollen in Arabidopsis and Lily

The methods of Wang et al. (2018a) and Sun and Pan (2008) with slight modifications were used for pollen quantification. When Arabidopsis plants were in full bloom, six white or recently bloomed flowers on the main stem were placed in a 1.5-ml centrifuge tube and dried in an oven at 60°C. The anthers were completely cracked, and the pollen was released; 1 mL of 200 g L^−1^ (NaPO_3_)_6_ solution was added, and the solution was oscillated for 2 min on a micro vortex mixer. Next, two drops of 2.5 μL of suspension from each of the three samples were placed on a glass slide. The number of pollen grains in the suspension was counted under the objective lens (4×) of an optical microscope (DM-6B, Leica, Germany). The number of pollen grains in each droplet was counted, and the average value of six droplets was recorded. The same procedures were repeated for three lily anthers at the full bloom stage, except that 8 mL of 200 g L^−1^ (NaPO_3_)_6_ solution was added after the anthers were dried.

Amount of pollen in lily = anther number of single flower × number of pollen grains per slide × 3200.

### Virus-Induced Gene Silencing of *LoMYB33*

To generate pTRV2-*LoMYB33*, a 300-bp gene-specific fragment was cloned into the pTRV2 vector using cDNA as a template. The primer pairs used to generate TRV vectors are shown in Supplementary Table 1. The recombinant plasmid was then transformed into *A. tumefaciens* GV3101. The bacterial solution was resuspended by the injection buffer (10 mM MgCl2, 100 mM 2-morpholinoethanesulfonic acid, 200 μM acetosyringone, pH = 5.8) (OD_600_ =1.0) and injected into “White heaven” leaves. Before injection, the mixture of 1:1 (v/v) *A. tumefaciens* culture containing pTRV1 and pTRV2, pTRV1, and pTRV2-*LoMYB33* was stored in the dark at room temperature for 3 h. Lily “White heaven” plants that had not yet produced flower buds were injected; the anthers were photographed during flowering and then collected for identification (Chen et al., 2020).

### Statistical Analysis

Microsoft Excel 2010 (Microsoft Corp., USA) and Statistical Product and Service Solutions v 612 17.0 (SPSS, USA) were used to analyze the data. Student's *t*-test was used to test for significant differences, and the threshold for statistical significance was *P* < 0.05. Student–Newman–Keuls test (*P* < 0.05) was used to compare means after ANOVA.

## Results

### LoMYB33 Is a GAMYB Member in Lily

**Figure 1:**
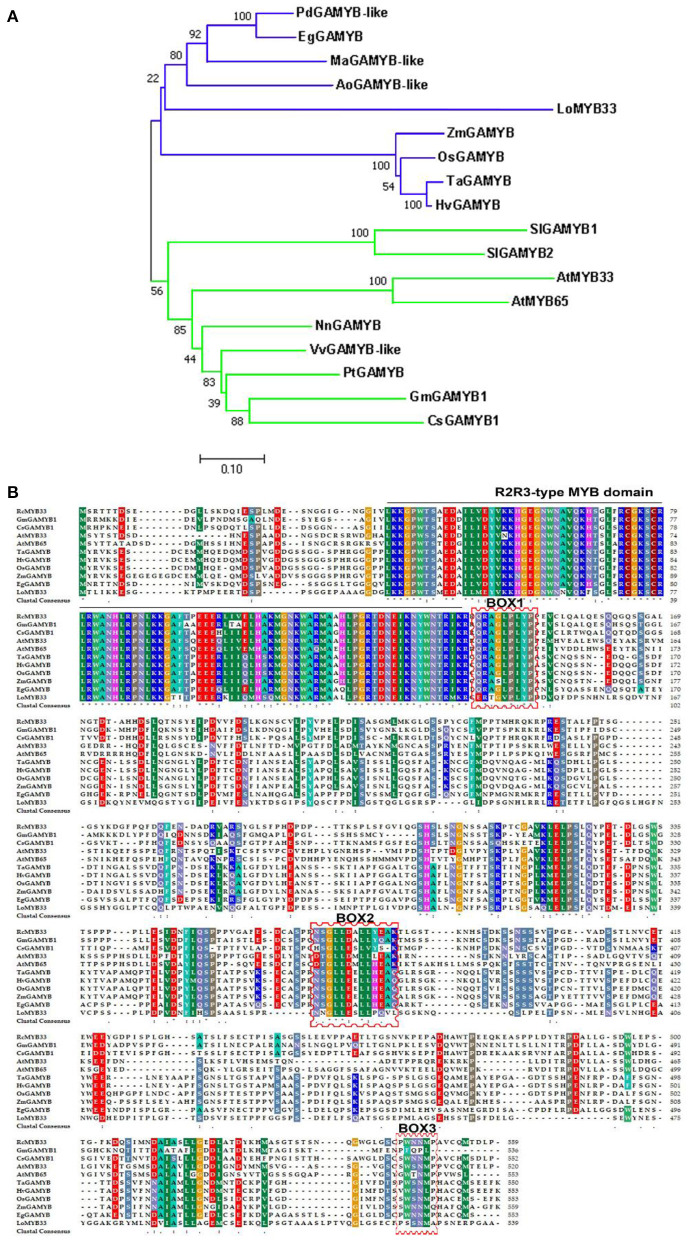
Amino acid sequence alignment, phylogenetic analysis, and bioinformatics analysis of LoMYB33. **(A)** Phylogenetic tree of LoMYB33 protein and GAMYB proteins from other plant species. Protein sequences of GAMYBs were taken from *Phoenix dactylifera* (PdGAMYB-like, XP_008792432.2), *Elaeis guineensis* (EgGAMYB, XP_010922970.1), *Lilium* spp. (LoMYB33), *Oryza sativa* (OsGAMYB, XP_015622335.1), *Triticum aestivum* (TaGAMYB, XP_037414515.1), *Arabidopsis* thaliana (AtMYB33, NP_001078537.1; AtMYB65, NP_001327042.1), *Rosa chinensis* (RcMYB33, XP_024166362.1), *Zea mays* (ZmGAMYB, NP_001241838.2), *Glycine max* (GmGAMYB1, NP_001304541.1), *Hordeum vulgare* (HvGAMYB, KAE8787635.1) and *Cucumis sativus* (CsGAMYB1, XP_004140923.1), *Musa acuminata* (MaGAMYB-like, XP_009398961.1), *Nelumbo nucifer*a (NuGAMYB, XP_010251854.1), *Populus tomentosa* (PtGAMYB-like, AZQ25444.1), *Vitis vinifera* (VvGAMYB-like, XP_034705115.1), *Asparagus officinalis* (AoGAMYB-like, XP_020253654.1), and *Solanum lycopersicum* (SlGAMYB1, Solyc01g009070; SlGAMYB2, Solyc06g073640). **(B)** Protein sequences of GAMYBs were from *Elaeis guineensis* (EgGAMYB, XP_010922970.1), *Lilium* spp. (LoMYB33), *Oryza sativa* (OsGAMYB, XP_015622335.1), *Arabidopsis thaliana* (AtMYB33, NP_001078537.1; AtMYB65, NP_001327042.1), *Rosa chinensis* (RcMYB33, XP_024166362.1), *Triticum aestivum* (TaGAMYB, XP_037414515.1), *Zea mays* (ZmGAMYB, NP_001241838.2), *Hordeum vulgare* (HvGAMYB, KAE8787635.1), and *Cucumis sativus* (CsGAMYB1, XP_004140923.1); the R2R3 domain is indicated by black lines, and the BOX motifs are indicated by red frames.

The *LoMYB33* ORF was 1,620 bp and encoded a protein with 539 amino acids. Phylogenetic analysis with 124 MYB family TFs from Arabidopsis indicated that LoMYB33 was closely related to AtMYB33 and AtMYB65 (Supplementary Figure 1), both of which are GAMYB family members (Millar and Gubler, 2005; Tsuji et al., 2006; Li et al., 2016). A BLAST search against the Arabidopsis TAIR database revealed that LoMYB33 was most closely related to AtMYB33 (Supplementary Figure 2); thus, this protein was named LoMYB33. A phylogenetic analysis of LoMYB33 with MYB members in rice revealed that it is more closely related to OsGAMYB (Supplementary Figure 3), suggesting that LoMYB33 is a member of the GAMYB family. The alignment of LoMYB33 with *Phoenix dactylifera, Elaeis guineensis, Oryza sativa*, Arabidopsis, *Rosa chinensis* and other GAMYB homologs revealed that LoMYB33 was clustered with the GAMYBs of *P. dactylifera* and *E. guineensis, Musa acuminata*, and *Asparagus officinalis*; *P. dactylifera, E. guineensis, M. acuminata, A. officinalis*, and lily are non-grass monocotyledonous plants (Figure 1A). Multiple protein alignment with the amino acid sequences of homologs from *E. guineensis, O. sativa*, Arabidopsis, *R. chinensis, Zea mays, Glycine max, Hordeum vulgare, Triticum aestivum, Solanum lycopersicum*, and *Cucumis sativus* showed that LoMYB33 contained the classical R2R3 domain of the R2R3-MYB family (Figure 1B), indicating that it belonged to the R2R3-MYB family. In addition, LoMYB33 contained three typical BOX1, BOX2, and BOX3 motifs of the GAMYB family. BOX1 is a QRaGLPxYPx (E/S) motif located near the C-terminal next to the R2R3 repeat DNA-binding domain (Kranz et al., 1998; Romero et al., 1998). BOX2 and BOX3 are also conserved regions of GAMYBs (Gocal et al., 2001). Protein alignment revealed that the BOX1, BOX2, and BOX3 motifs of LoMYB33 were not completely conserved, as some amino acid substitutions were detected; a similar pattern was observed for GmGAMYB1, GsGAMYB1, AtMYB65, and ZmGAMYB (Figure 1B).

### *LoMYB33* Is Specifically Expressed in Anther

**Figure 2:**
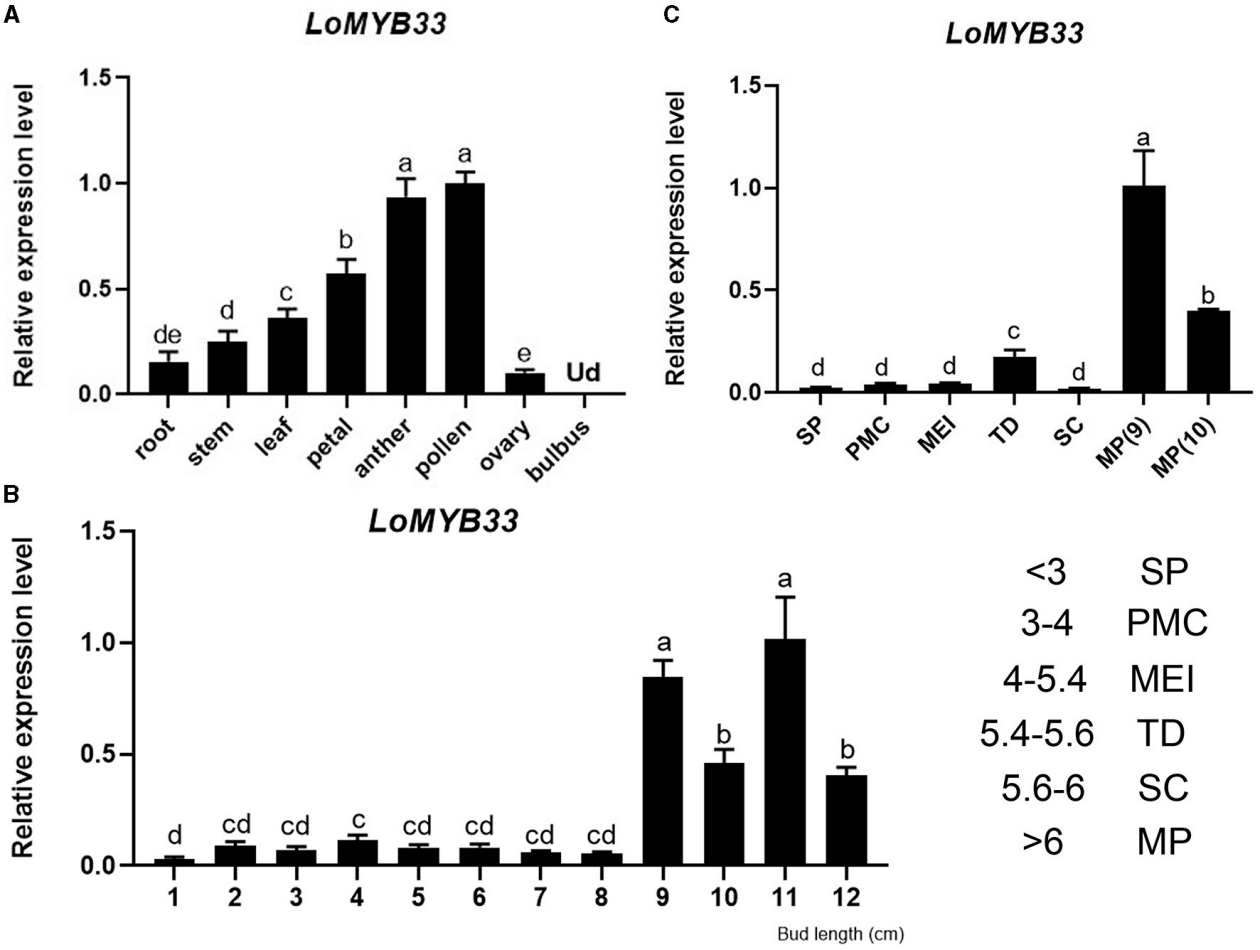
Expression analysis of *LoMYB33*. (**A**) Relative expression of *LoMYB33* in different organs (9-cm flower bud) by RT-qPCR analysis. (**B**) Relative expression of *LoMYB33* in anthers of 1 to 12-cm flower buds. (**C**) Relative expression of *LoMYB33* in pollen from sporogenous cells to mature pollen. Data are mean ± SD of three independent experiments. Different letters indicate significant differences among these lines (Student–Newman–Keuls test, *P* < 0.05). SP, sporulation period; PMC, pollen mother cell stage; MEI, meiotic stage; TD, tetrad stage; SC, single-core stage; MP, mature pollen. Ud, Undetermined. The number indicates the length of the flower bud of “Siberia” lily, the unit is cm.

We detected the expression of *LoMYB33* in the bulbus, root, stem, leaf, petal, anther, and ovary of lily by RT-qPCR (Figure 2A). The expression of *LoMYB33* was significantly higher in male organs such as anther and pollen than in female organs; *LoMYB33* expression was also higher in male organs than in vegetative organs (Figure 2A). The expression of *LoMYB33* was significantly higher in late developmental stages than in early developmental stages; *LoMYB33* expression was highest in anthers collected from 11 cm flower buds, followed by buds of 9, 10, and 12 cm (Figure 2B). The expression of *LoMYB33* was highest in mature pollen, especially during the early stage of pollen maturation (Figure 2C). In addition, there is a small expression peak in the tetrad stage of anther development (Figure 2C). These results indicated that *LoMYB33* was mainly expressed in the late development stages of anthers; its transcripts were most highly accumulated in pollen, and it might also function in the tetrad stage of anther.

### The Promoter Activity of *LoMYB33* Is Strongly Activated in the Anthers and Pollen

**Figure 3:**
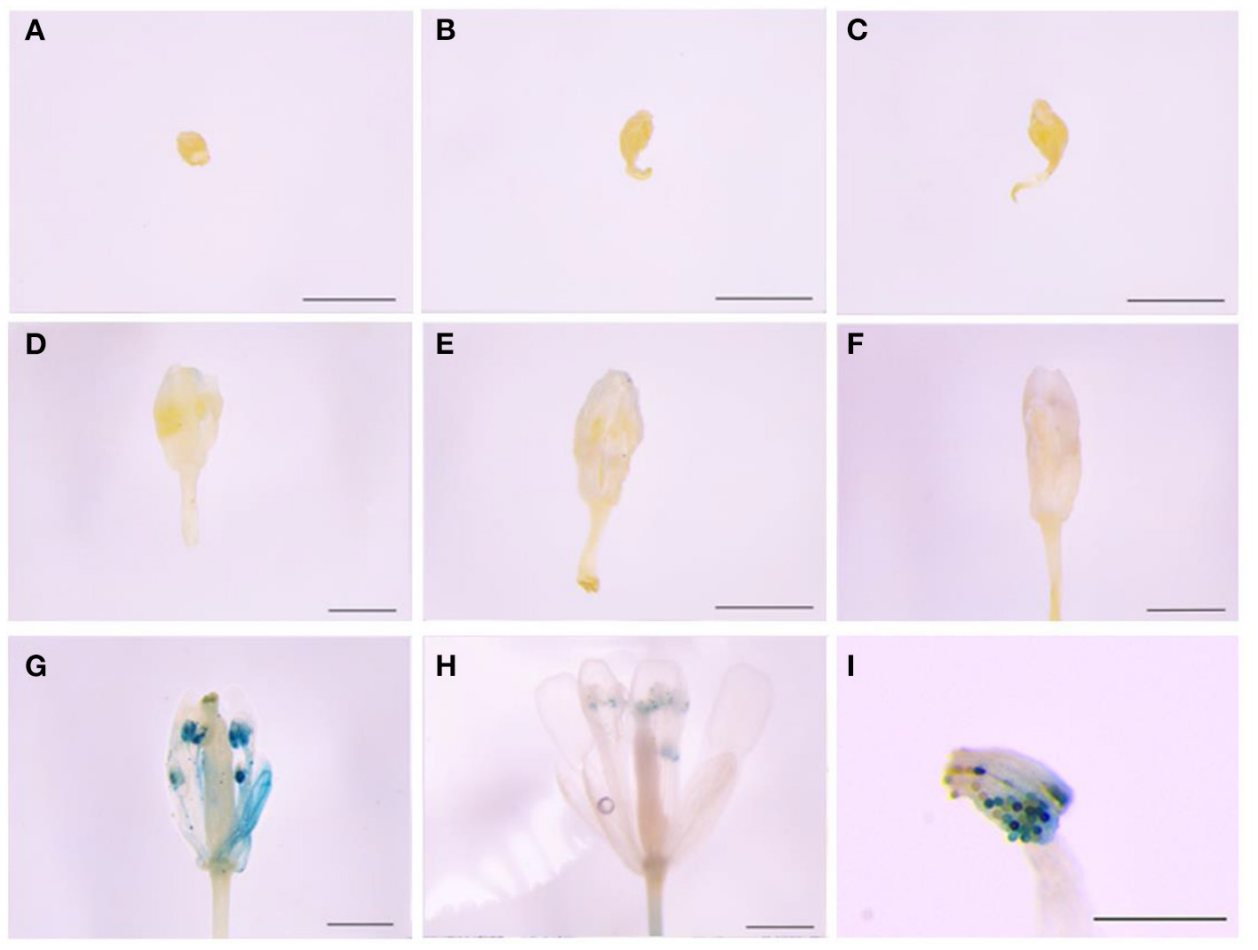
Analysis of *LoMYB33* promoter activity. (**A–H**) show the results of the activity analysis of GUS for stages 1–13. **(A)** Sporulation stage (stage 2); **(B)** mother cell stage (stage 5); **(C)** start of the meiotic period (stage 6); **(D)** microspore stage (stage 8); **(E)** spore stage in which spores are small (stage 9); **(F)** microspore stage (stage 10); **(G)** mature pollen stage (stage 12); **(H)** mature pollen stage (stage 13); and **(I)** GUS staining of pollen in stage 12 flowers. Three independent experiments were performed, and one representative picture is shown. Bars are 1 mm.

The 465-bp promoter of *LoMYB33* was obtained and analyzed using PlantCARE online software (Supplementary Figure 4). The promoter contained 1 ARE, 5 CAAT-boxes, 2 GC-motifs, 1 TATA-box, and 2 TCCC-motifs. These elements were related to the photoresponse and anaerobic induction. The *GUS* gene driven by the *LoMYB33* promoter was transformed into Arabidopsis; GUS histochemical staining analysis revealed that the *LoMYB33* promoter was activated in the later stages of Arabidopsis anther development but was not activated in the early stages (Figures 3A–H), a pattern consistent with its expression in lily anthers (Figure 2). As expected, high GUS activity was observed in mature pollen (Figure 3I), which indicated that the promoter of *LoMYB33* was highly activated in pollen.

### LoMYB33 Is a Cytoplasm-Nucleus Localization Protein

**Figure 4:**
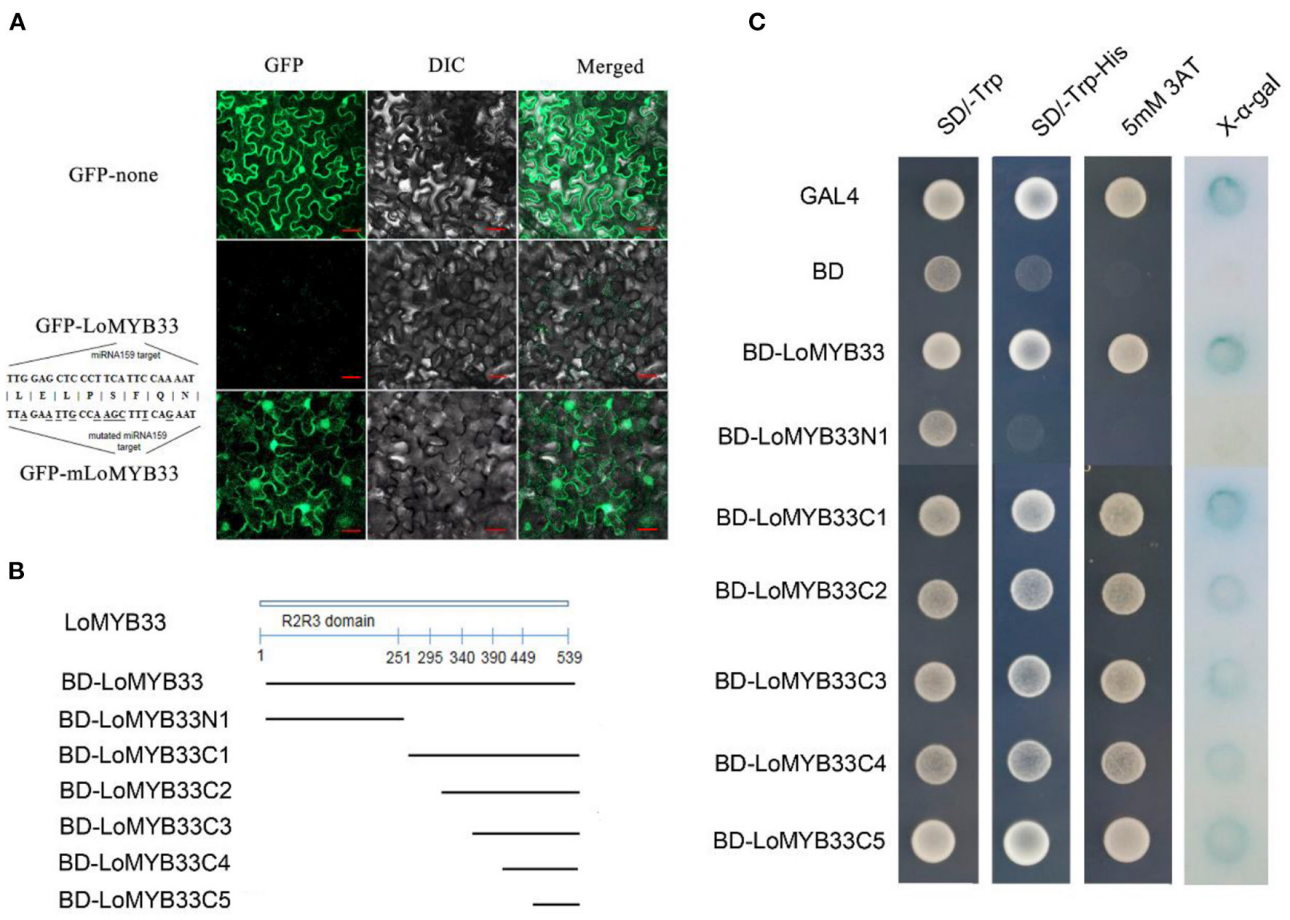
Analysis of LoMYB33 transcriptional activation activity and subcellular location. **(A)** The GFP (up) protein, LoMYB33-GFP (middle), and mLoMYB33-GFP (down) were transiently expressed in tobacco leaves. **(B)** The lines below the LoMYB33 diagram indicated the different regions that were used in the transactivation assay. All constructs fused with the GAL4 DNA-binding domain (BD) were inserted into the expression vector pGBKT7. **(C)** Transactivation activity of different constructs of LoMYB33 in yeast cells. The positive control was GAL4, and the negative control was BD vector. The SD-Trp medium (left panel) was used to detect transformation, the SD-Trp/His medium (middle panel) was used to examine transformant growth, and X-α-gal staining was used to detect β-galactosidase activity of transformed yeast cells (the right panel).

The GFP signal of LoMYB33-GFP was not observed, but the GFP protein signal was observed following transient expression of the GFP fusion protein in tobacco leaves. Analysis of the gene sequence of *LoMYB33* revealed that *LoMYB33* might be a target of miRNA159; this was confirmed in a previous study of lily (Gao et al., 2020). In Arabidopsis, the *GAMYB* genes *AtMYB33* and *AtMYB65* are also miRNA159 targets (Rhoades et al., 2002; Allen et al., 2007). Analysis of the miRNA159 target sites of *LoMYB33* revealed that the conserved TTGGAGCTCCCTTCATTCCAAAAT sequence of *LoMYB33* might be the target sequence, which is located from 966 to 990 bp. Therefore, we speculated that wild-type *LoMYB33* might be cut by miRNA159 in the tobacco cells. The miRNA159 target sites of *LoMYB33* were then mutated to *mLoMYB33*, which was also transiently expressed in tobacco leaves. A large number of fluorescent signals appeared, and mLoMYB33-GFP was located in the nucleus and cytoplasm (Figure 4A), suggesting that LoMYB33 is a cytoplasm-nucleus localization protein.

### The C-Terminal of LoMYB33 Contributes to Its Transactivation Ability

The full length and different portions of LoMYB33 were fused to the pGBKT7 vector and then transformed into the yeast strain AH109 (Figure 4B). Full-length LoMYB33 had transcriptional activation activity; all C1–C5 fragments showed transcriptional activation activity but not N1 (Figure 4C). Therefore, the transcriptional activation domain of LoMYB33 was within 90 amino acids of the C-terminal.

### GA Treatment Activates the Expression of *LoMYB33* in Anthers

**Figure 5:**
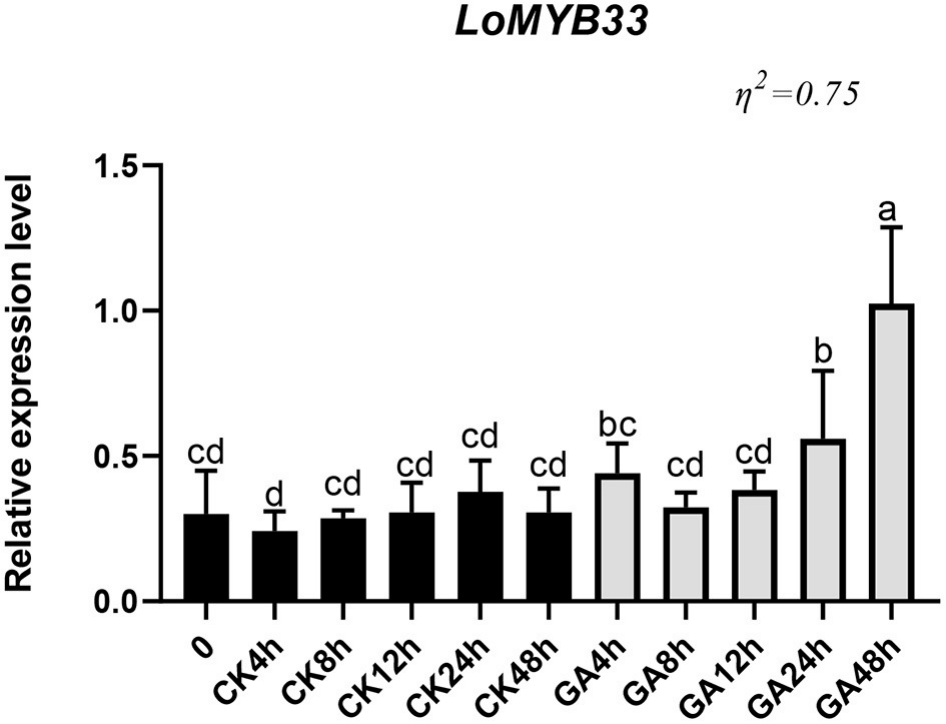
Expression of *LoMYB33* in lily anthers after GA treatment. CK was the control group, and GA was the experimental group; the expression of *LoMYB33* was determined at 0, 4, 8, 12, 24, and 48 h after GA treatment. Data are mean ± SD of three independent experiments. Different letters indicate significant differences among these lines (Student–Newman–Keuls test, *P* < 0.05).

To study the effect of GA on the expression of *LoMYB33* in anthers, lily “Siberia” plants showing normal growth were treated with GA_3_, and the expression of *LoMYB33* was determined at several time points after treatment. The expression of *LoMYB33* after GA_3_ treatment was up-regulated relative to the control (Figure 5). The expression of *LoMYB33* in the control changed little following treatment, but the expression of *LoMYB33* in the experimental group changed significantly after treatment; the expression of *LoMYB33* was highest after 48 h of GA_3_ treatment, followed by 24 h of GA_3_ treatment (Figure 5). These results indicated that GA_3_ treatment could activate the expression of *LoMYB33* in anthers.

### Overexpression of *LoMYB33* Causes Partial Male Sterility

**Figure 6:**
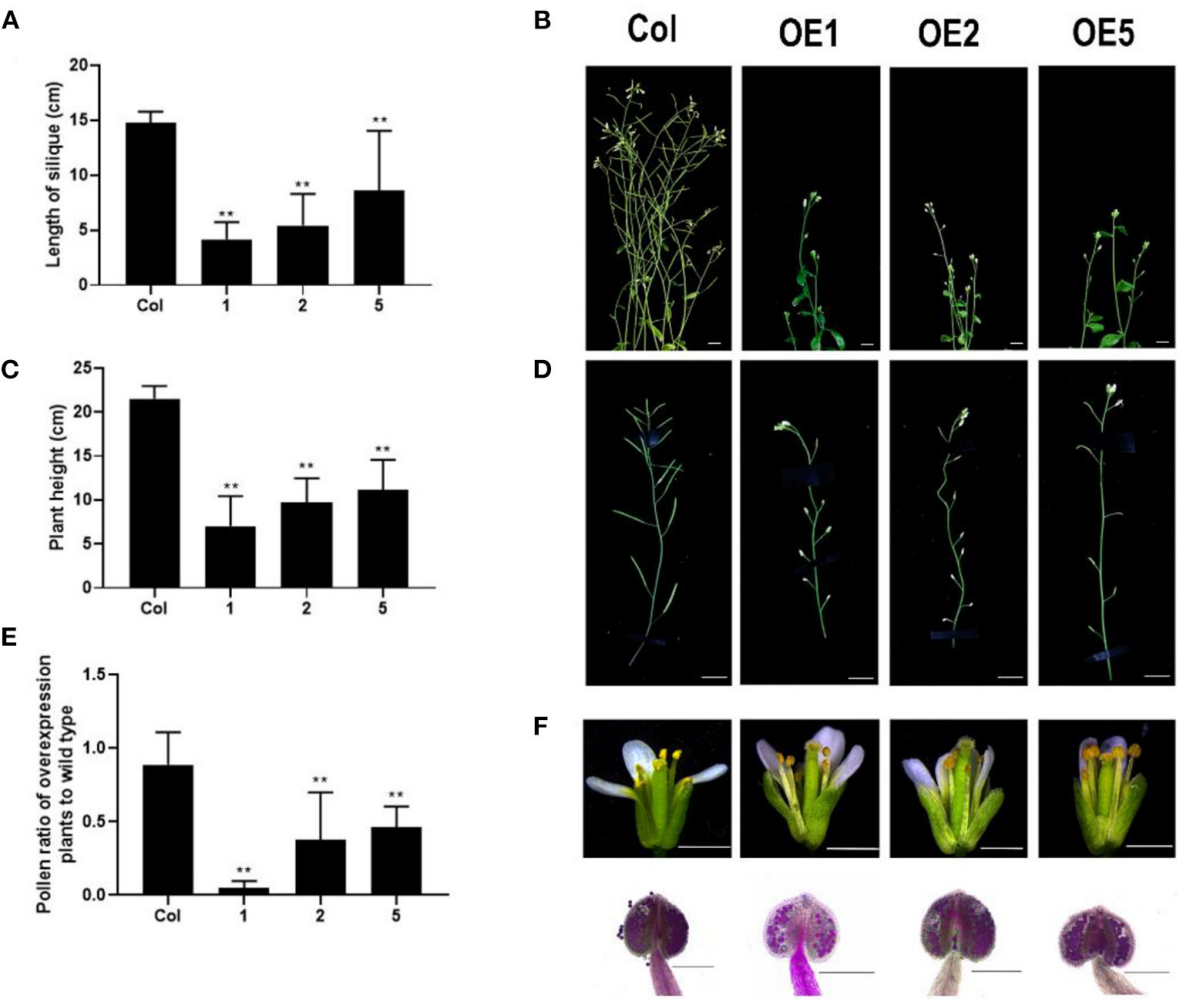
Morphological differences between *LoMYB33-*overexpressing and wild-type Arabidopsis plants. **(A)** Statistics of plant height of Col-0 and three overexpression lines (OE1, OE2, and OE5); **(B)** The main inflorescence of Col-0, OE1, OE2, and OE5; **(C)** Statistics of pod length of Col-0 and three overexpression lines. **(D)** Comparison of silique growth of Col-0, OE1, OE2, and OE5; **(E)** The relative ratio of the amount of pollen of three LoMYB33-OE lines and Col-0; **(F)** flowers at stage 13 of Col-0, OE1, OE2, and OE5. Bars are 1 mm. The anthers of Col-0, OE1, OE2, and OE5 stained by Alexander stain; the bars are 100 μM. At least three independent replicates were used for phenotypic identification; a representative result is presented. Data are mean ± SD of the tested plants. Data are mean ± SD of three independent experiments (*t*-test, **P < 0.01).

To explore the function of LoMYB33, *LoMYB33*-overexpressing Arabidopsis plants were generated, and three independent overexpression (OE) lines were selected by RT-PCR (Supplementary Figure 5). RT-qPCR was performed on the inflorescence of wild-type plants and three *LoMYB33*-OE lines, and high expression levels of *LoMYB33* were observed in the three overexpression lines. The expression levels of some pollen development-related genes, *AtMYB33, AtMYB65, AtCYP703A2, AtCYP704B1*, and *AtACOS5*, were significantly increased (Supplementary Figure 6). In addition, the normal growth of *LoMYB33*-OE plants was inhibited compared with wild-type plants, as these plants were shorter and later flowering (Figures 6A,B). The siliques of transgenic plants were significantly shorter compared with wild-type plants (Figures 6C,D), suggesting that the fertility of transgenic plants was reduced. Alexander staining revealed that the amount of pollen was lower in the three transgenic lines than in wild-type plants (Figure 6E). Transgenic plants contained less pollen in each anther compared with wild-type plants (Figure 6F), which might explain the reduction in fertility of transgenic plants.

**Figure 7:**
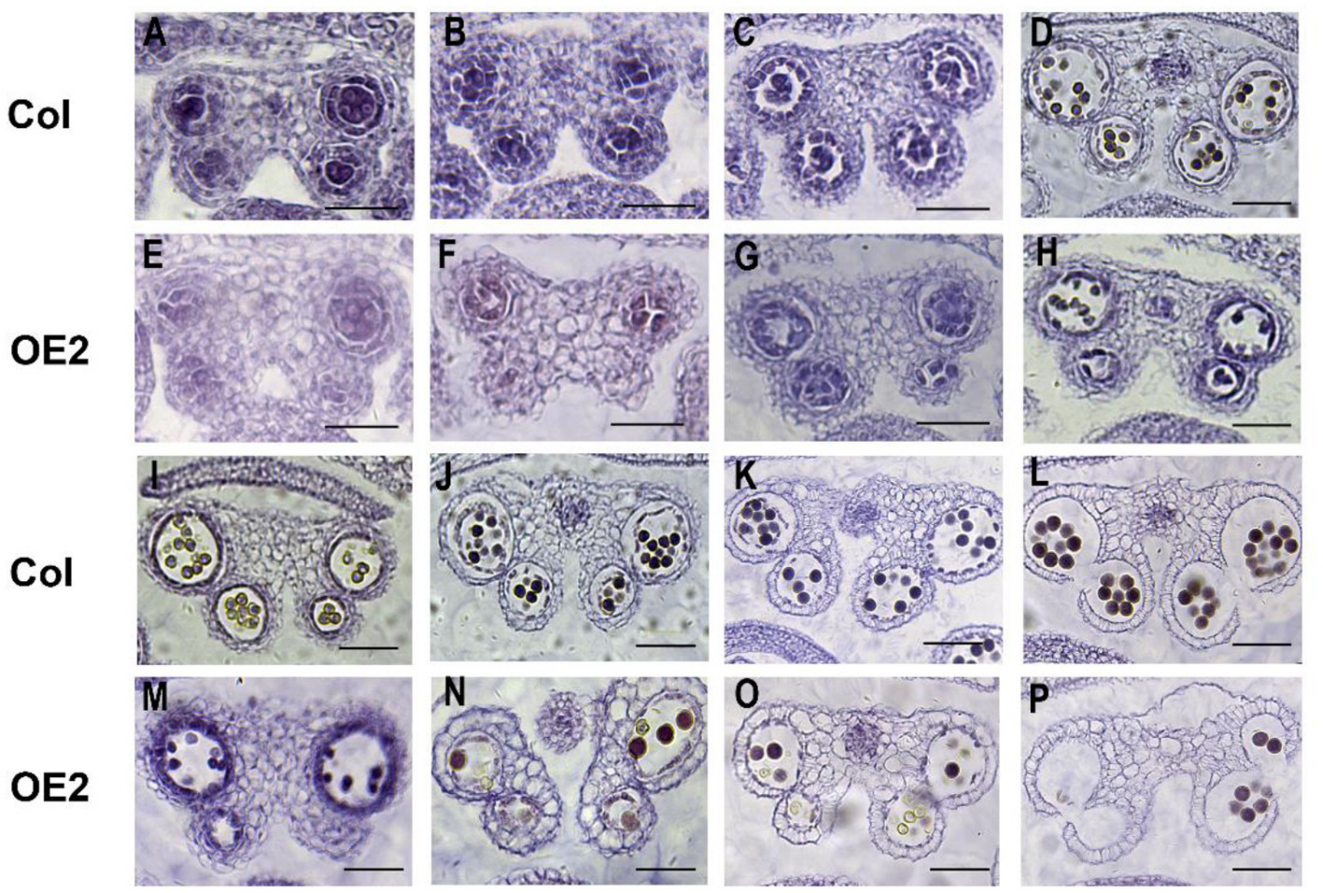
Cytological observations of different developmental stages of wild-type and *LoMYB33*-overexpressing Arabidopsis anthers. (**A**–**D**) Paraffin sections of anthers at stage 5–8 of wild-type plants. (**E**–**H**) Paraffin sections of anthers at stage 5–8 of OE2. (**I**–**L**) Paraffin sections of anthers at stages 9–14 of wild-type plants. (**M**–**P**) Paraffin sections of anthers at stage 9–14 of OE2. Three independent experiments were performed, and one representative picture is shown. Bars are 100 μm for stage 5–7. Bars are 50 μm for stage 8–14.

To further understand the effect of *LoMYB33* overexpression on anther development, the anther development of wild-type and *LoMYB33*-OE Arabidopsis plants was observed in paraffin sections. The anther development of Arabidopsis was observed from stage 5 to 14 (Figure 7). When microspore mother cells underwent meiosis in four chambers and produced tetrads of haploid microspores in stage 7, the anther development of transgenic plants was abnormal; there was no tapetum in the anther compartment, and the middle layer cells did not degrade; and the development of the tapetum in OE-2 was delayed (Figures 7A–C,E–G). From stage 8 to 14, the pollen number of transgenic plants was lower than that of wild-type plants (Figures 7D,H,I–P). These results indicated that the overaccumulation of *LoMYB33* might damage the normal development of pollen and anthers, suggesting that LoMYB33 might play a key role in pollen development; thus, an appropriate level of *LoMYB33* expression is essential for normal development.

### Silencing of *LoMYB33* Reduces the Amount of Pollen in Lily

**Figure 8:**
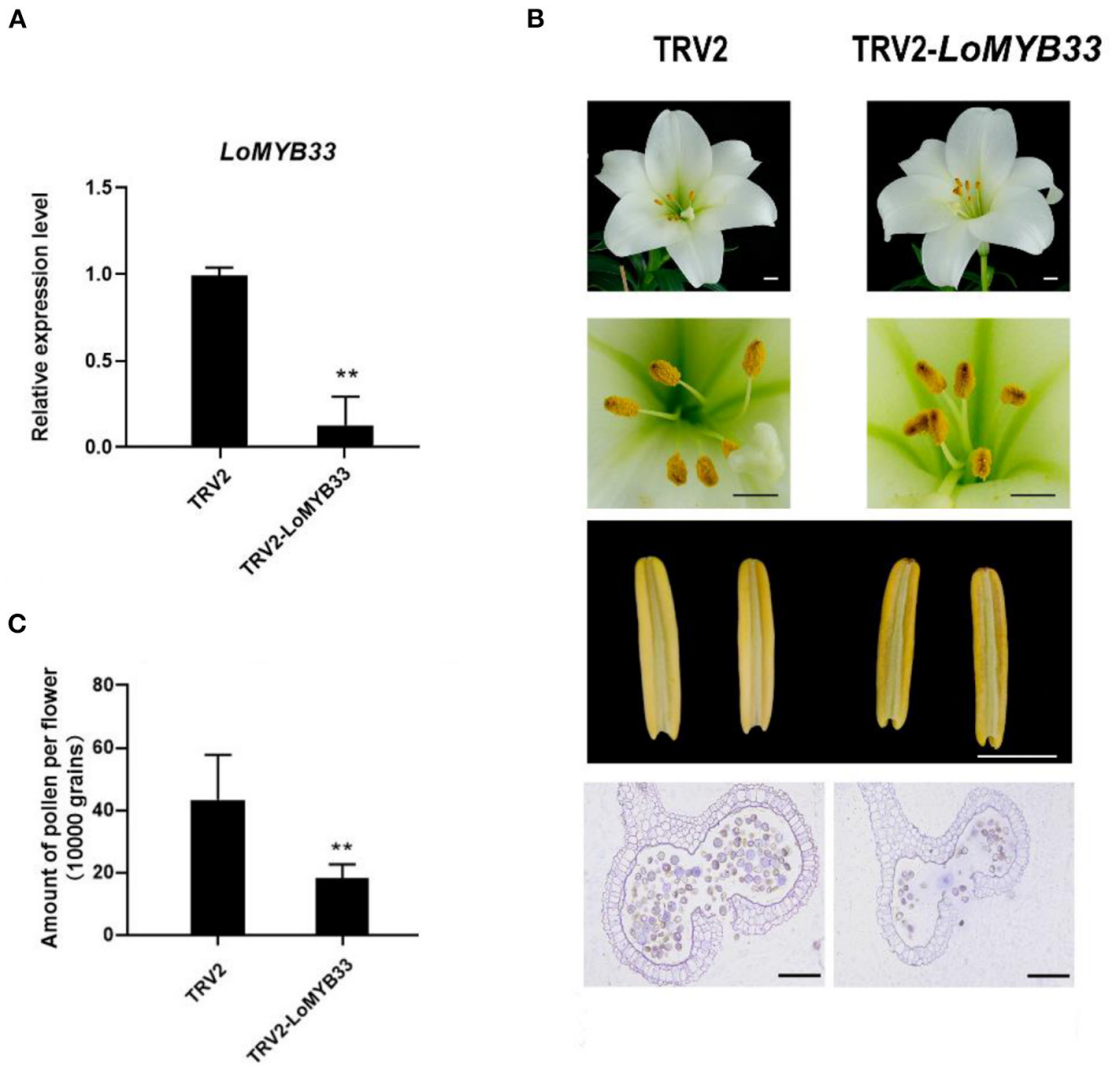
Silencing of *LoMYB33* in lily anthers by VIGS. (**A**) *LoMYB33* expression was determined in *LoMYB33*-silenced and TRV-control plants by RT-qPCR. Data are mean ± SD of three independent experiments (*t*-test, **P < 0.01). (**B**) Morphologies of flowers and anthers in TRV2 and TRV2-LoMYB33 lilies. Three independent experiments were performed, and one representative picture is shown. (**C**) Differences in the amount of pollen between *LoMYB33*-silenced and TRV-control plants were analyzed. Bars are 1 cm and 100 μm. Data are mean ± SD of three independent experiments (*t*-test, **P < 0.01).

To determine whether *LoMYB33* is involved in the development and formation of pollen in lily, VIGS of *LoMYB33* was carried out in lily “White heaven” because its growth and development cycle is much shorter compared with other cultivars. Specific primers in the non-conservated region of *LoMYB33* were designed to construct the TRV2-*LoMYB33* silencing vector. RT-PCR showed that the TRV vectors had successfully spread to the anthers of TRV2-*LoMYB33* lily lines (Supplementary Figure 7), and *LoMYB33* expression in anthers was significantly decreased after silencing *LoMYB33* (Figure 8A). The shape of lily flowers in TRV-control and *LoMYB33*-silenced lines did not differ, but the anthers became more withered in the *LoMYB33*-silenced lines. The amount of pollen was significantly reduced in these *LoMYB33*-silenced anthers compared with the TRV2-control (Figures 8B,C). These findings indicated that *LoMYB33* might play an important role in anther development and pollen formation in lily.

## Discussion

### LoMYB33 Belongs to the GAMYB Family in Lily

Some R2R3-MYB members encoded by *GAMYB* genes have been shown to act as transcriptional activators of the GA signal pathway and play various roles in plants. In Arabidopsis, GAMYBs include three members: *AtMYB33, AtMYB65*, and *AtMYB101*. *AtMYB33* and *AtMYB65* are co-expressed in many tissues and have redundant functions. The *myb33/myb65* double mutant shows defective anther development (Millar and Gubler, 2005). *AtMYB101* expression is restricted to the subapical pith cells of both vegetative and flowering plants and to the hypocotyl hook and may be involved in GA-regulated stem elongation (Gocal et al., 2001). LtGAMYB plays an important signaling role in the flowering of ryegrass (Gocal et al., 1999). *TaGAMYB* expression is related to the length of wheat internodes (Chen et al., 2001). In germinated rice seeds, OsGAMYB induced the biological expression of α*-amylase* genes in the aleurone layer (Sutoh and Yamauchi, 2003). In tobacco, miR159 inhibition increases the expression of *NtGAMYB*, up-regulating disease resistance genes and enhancing resistance to *Phytophthora* (Zheng et al., 2020). In this study, we cloned and identified *LoMYB33* from lily and showed that it may play a role in the development of anthers and pollen. Protein sequence analysis showed that LoMYB33 belonged to the R2R3-MYB family and that it contained three typical motifs of the GAMYB family (Figure 1) (Dubos et al., 2010). Phylogenetic analysis with Arabidopsis MYB members revealed that LoMYB33 is closely related to AtMYB33 and AtMYB65 (Supplementary Figure 1), both of which are GAMYB members. AtMYB33 and AtMYB65 have redundant functions in regulating anther and pollen development in Arabidopsis (Millar and Gubler, 2005). In rice, the homolog OsGAMYB also regulates the development of anthers (Aya et al., 2009). Based on the sequence alignment and phylogenetic analysis, we speculate that LoMYB33 is a GAMYB member in lily and that it may also play a role in anther development. According to previous studies, many species possess more than one member in the GAMYB family, such as Arabidopsis, tobacco, tomato, and rice, and these GAMYB members show redundant and special functions in different physiological processes (Gocal et al., 2001; Millar and Gubler, 2005; Tsuji et al., 2006; Gong and Bewley, 2008; da Silva et al., 2017; Zheng et al., 2020). In this study, *LoMYB33* exhibited an expression pattern that was inconsistent with many reported *GAMYBs* of model plants (Figure 2), which suggested that the members of GAMYB family might be functionally differentiated in lily and that there might be other GAMYB members in lily.

### LoMYB33 Shows Both Conserved and Divergent Functions and May Primarily Function in the Late Stage of Anther Development

Transcription activation activity analysis revealed that LoMYB33 had transactivation activity, the transactivation domain of R2R3-MYBs is generally distributed at the C-terminal (Zhao et al., 2017); as in previous studies, the C-terminal contributed to the transactivation ability of LoMYB33 (Figure 4).

Subcellular localization analysis of LoMYB33 was carried out, but no GFP fluorescence signal was observed when the wild-type ORF of *LoMYB33* was used to construct the recombinant plasmid. Based on studies in model plants, GAMYBs are regulated by miRNA159 (Palatnik et al., 2007), miRNA159 is regulated by GA and regulates anther development by cutting GAMYB mRNA, and the target sites are conserved in different species (Achard et al., 2004; Csukasi et al., 2012; Zheng et al., 2020). Therefore, we mutated the predicted target sites of miRNA159 in *LoMYB33* for subcellular localization analysis; as expected, GFP fluorescence was observed and located in the nucleus and cytoplasm (Figure 4), which suggests that *LoMYB33* might also be regulated by the conserved miRNA159-GAMYB pathway in lily as in other plants.

After GA treatment of lily, the expression of *LoMYB33* in anthers increased significantly (Figure 5), which indicated that GA could activate the expression of *LoMYB33* in anthers. In barley, the expression of *HvGAMYB* was also activated by GA treatment (Gubler et al., 1995, 2002). Exogenous GA treatment resulted in the increased expression of *AtMYB33* in Arabidopsis (Achard et al., 2004). Our results showed that *LoMYB33* was activated by GA and thus that *LoMYB33* might be a GA signal transduction factor similar to AtMYB33. However, additional research is needed to determine how LoMYB33 responds to GA signals.

According to the results of the RT-qPCR analysis in this study, *LoMYB33* was the most highly expressed in pollen, followed by the anthers (Figure 2). *LoMYB33* was highly expressed in the late anther development stages compared with the early anther development stages, which suggested that LoMYB33 might function in the late anther developmental stages. Contrary to expectation, *LoMYB33* accumulated in mature pollen, especially in pollen collected from the anthers of 11-cm flower buds (Figure 2). *LoMYB33* was also significantly higher in immature pollen at the tetrad stage than in other early stages of pollen development, although there was no difference in anthers from the same stage (Figure 2). High activity of the *LoMYB33* promoter was detected in anthers at stages 12 to 13 of transgenic Arabidopsis plants, especially in mature pollen (Figure 3), which was consistent with the pattern of expression of *LoMYB33* in lily anthers. In rice, the expression level of *OsGAMYB* was lowest in the mature pollen stage, and highest in the tetrad and mononuclear microspore stages (Aya et al., 2009). *HvGAMYB* in barley and both *AtMYB33* and *AtMYB65* in Arabidopsis are all weakly expressed in mature pollen grains (Murray et al., 2003; Kaneko et al., 2004; Millar and Gubler, 2005; Aya et al., 2009). The expression of *LoMYB33* is inconsistent with the expression patterns of these genes. In cucumber, *CsGAMYB1* is most highly expressed during the critical period when the stamen primordium and carpel primordium are initiated; it is also highly expressed in the mature pollen grains of male cucumber flowers (Zhang et al., 2014). These results indicate that the expression of *LoMYB33* and its homologs may involve different conserved regulatory mechanisms. The function of LoMYB33 in the tetrad stage may be similar to that of OsGAMYB, which regulates the development of anthers by controlling the development of the tapetum (Figure 7) (Kaneko et al., 2004). In addition, the high expression of *LoMYB33* in later stages may affect pollen maturation and germination. The presence of a certain amount of GA in pollen is required for pollen germination (Meeuse et al., 1976). GA also affects the biosynthesis of flavanols, which are important components in mature pollen (Koornneef and Veen, 1980; Cheng et al., 2009). LoMYB33 may participate in the GA signaling pathway in anthers and pollen and affect the germination and maturation of pollen.

In Arabidopsis, *AtMYB33* is strongly expressed in the developing young anther chambers and weakly expressed in the pollen grains (Gocal et al., 2001; Millar and Gubler, 2005). The *AtMYB33* promoter activity is strong in tapetum cells but weak in other anther wall layers and microspores (Aya et al., 2009). These results indicate that the activation pattern of *LoMYB33* promoter in lily differed from that in Arabidopsis and rice, which suggests that LoMYB33 might be involved in pollen maturation and exine formation.

### LoMYB33 Can Regulate Anther and Pollen Development in Transgenic Arabidopsis Plants

Overexpression of *LoMYB33* in Arabidopsis significantly inhibits plant growth, results in late flowering, and decreases fertility (Figure 6). GAMYB homologs have been reported to regulate flowering time in Arabidopsis, ryegrass, barley, and rice (Blazquez et al., 1998; Blazquez and Weigel, 1999; Gocal et al., 1999; Murray et al., 2003; Kaneko et al., 2004). GAMYB is a promoter of plant flowering induction (Gocal et al., 1999), but *SlMYB33* overexpression in tomato delays flowering (Zhang et al., 2020). Transgenic Arabidopsis plants with *LoMYB33* are short and have short internodes; *GmGAMYB* overexpression accelerates flowering in soybean and increases plant height (Yang et al., 2021). In Arabidopsis, overexpression of resistant types of *AtMYB33* and *AtMYB65* with mutated miRNA159 target sites results in short plants and male sterility. However, no change in plant growth was noted in transgenic Arabidopsis plants overexpressing wild-type *AtMYB33* or *AtMYB65* because miRNA159 cleaves AtMYB33 and AtMYB65 in vegetative tissues (Palatnik et al., 2003; Li et al., 2016). Overexpression of wild-type *LoMYB33* retarded plant growth (Figure 6). In poplar, no phenotypic changes were observed following overexpression of the miRNA159 target gene *PtrMYB012*; however, upward curling of the leaves, dwarfism, and male sterility were observed in *PtrMYB012-*transgenic Arabidopsis. Thus, *PtrMYB012* may be completely degraded by miRNA159 in poplar but not in Arabidopsis (Kim et al., 2018). We speculated that LoMYB33 might not be completely degraded by Arabidopsis miRNA159 because of the species specificity of miRNA159.

In transgenic Arabidopsis plants, the expression of *AtCYP703A2, AtCYP704B1*, and *AtACOS5* was significantly increased (Supplementary Figure 4), which suggested that these genes might be located downstream of *LoMYB33* and that LoMYB33 activated their expression. In rice, OsGAMYB binds to the promoter of *OsCYP703A3* through its MYB domain to activate its expression for the formation of the Ubisch body and the pollen outer wall (Aya et al., 2009). Acyl-CoA synthetase ACOS5 (a cytochrome P450 hydroxylase family member), CYP703A2, and CYP704B1 are all involved in pollen exine synthesis (Wang et al., 2018b), which suggests that LoMYB33 might play key roles in the maturation of pollen and the formation of pollen walls. In transgenic lines, the expression of *AtMYB33* and *AtMYB65* was also up-regulated, which might stem from their self-activating activity. The expression of *AtMYB33, AtMYB65*, and *AtCYP704B1* is independent of *LoMYB33* expression, and the expression pattern of *AtCYP703A2* is opposite that of *LoMYB33* (Wu et al., 2019; Ding et al., 2021). We speculate that this might be explained by a negative feedback regulation mechanism involving *LoMYB33*, wherein the high expression of *LoMYB33* activates the feedback pathway to control the expression of these genes at a level appropriate for anther development. Our results indicate that LoMYB33 had a dose-regulating effect, as an appropriate expression level is required to ensure the normal development of the anthers (too high or too low expression leads to abnormal anther development).

When microspore mother cells undergo meiosis in four chambers and produce tetrads of haploid microspores in the 7^th^ stage of anther development, anther development in *LoMYB33*-overexpressing plants was abnormal because the tapetum in the anther chamber and the cells in the middle layer were not degraded. In the tetrad stage of wild-type plants, the mesothelium cells had degraded, and the tapetum cells began to degenerate (Figure 7). From stage 8 to 14, the pollen number was lower in transgenic plants than in wild-type plants, and the morphology of some microspores or pollen was abnormal (Figure 7). The tapetum is the innermost layer of the anther wall, and it plays an important role in the development of microspores and pollen (Stevens and Murray, 1981; DeGuzman and Riggs, 2000; Taylor et al., 2010). In male sterile lines, pollen abortion is tied to tapetum abnormalities. The timely control of tapetal programmed cell death is essential for pollen maturation (Uzair et al., 2019). In Arabidopsis *myb33*/*myb65* double mutant anthers, the tapetum experiences hypertrophy at the pollen mother cell stage, which leads to the termination of pollen development before meiosis (Millar and Gubler, 2005). In rice, the abnormal enlargement of tapetum cells and microspore collapse occur in *gamyb-2* mutants at the mature pollen stage, which stemmed from the inability of tapetum cells to undergo programmed cell death (Aya et al., 2009). *LoMYB33* overexpression may lead to male sterility by disturbing normal tapetal development. OsGAMYB is a major transcriptional regulator of meiosis and early tapetum and pollen development in rice (Ko et al., 2021). The tapetum of *gamyb-4* and *gamyb-5* mutants elongated abnormally during the late stage of anther development and occupied the entire anther chamber, which resulted in the male sterility of rice (Liu et al., 2010).

### LoMYB33 May Be a Useful Candidate Gene for the Pollen-Free Breeding of Lily

The flower shape of *LoMYB33*-silenced lines was not altered relative to wild-type plants, but the anthers became shriveled, and the amount of pollen significantly decreased (Figure 8), which is consistent with the findings of previous studies in Arabidopsis and rice. Mutations of *AtMYB33* and *AtMYB65* in Arabidopsis and *GAMYB* in rice cause male sterility because of a lack of pollen production (Millar and Gubler, 2005; Liu et al., 2010). *LoMYB33* overexpression in Arabidopsis also leads to pollen reduction; over-accumulation of *LoMYB33* resulted in more severe pollen reduction (Figure 6), indicating that both high and low levels of *LoMYB33* lead to a serious decline in fertility. Transgenic barley plants overexpressing *HvGAMYB* may also cause male fertility, and the severity of the phenotype depends on the level of *HvGAMYB* accumulation (Murray et al., 2003). We speculate that the overexpression effects of *LoMYB33* may also be dose-dependent. Genes related to pollen development, such as *AtMYB24*, show a similar pattern; overexpression of *MYB24* cannot restore *opr3* stamen development, and proper overexpression of *MYB24* can restore stamen development and male fertility. The expression level of *MYB24NT* is related to male sterility (Huang et al., 2017). Overall, these findings suggest that LoMYB33 may be involved in both tapetum development and pollen formation. *LoMYB33* may provide a useful candidate gene for the pollen-free breeding of lily.

## Data Availability Statement

The datasets presented in this study can be found in online repositories. The names of the repository/repositories and accession number(s) can be found at: GenBank, MZ666106.

## Author Contributions

NT and ZW conceived and designed the experiments. XL, JF, GY, and LH performed the experiments under the supervision of NT. DZ provided technical help. XL and ZW analyzed the data and wrote the manuscript. All authors read and approved the final version of the manuscript.

## Funding

This work was supported by a Project Funded by the Priority Academic Program Development of Jiangsu Higher Education Institutions, the earmarked fund for germplasm resources of Nanjing Agricultural University (KYZZ2019020), the High Level Talent Project of the Top Six Talents in Jiangsu (Grant No. NY-077), and the Programs for New Century Excellent Talents in Universities, Ministry of Education of China (NCET-11-0669).

## Conflict of Interest

The authors declare that the research was conducted in the absence of any commercial or financial relationships that could be construed as a potential conflict of interest.

## Publisher's Note

All claims expressed in this article are solely those of the authors and do not necessarily represent those of their affiliated organizations, or those of the publisher, the editors and the reviewers. Any product that may be evaluated in this article, or claim that may be made by its manufacturer, is not guaranteed or endorsed by the publisher.
