# Supplementary material for: A Novel R2R3-MYB Gene LoMYB33 From Lily Is Specifically Expressed in Anthers and Plays a Role in Pollen Development
Source: Front Plant Sci. 2021 Sep 23;12:730007. doi: 10.3389/fpls.2021.730007 (PMC8495421; doi:10.3389/fpls.2021.730007)
Supplement: Supplementary file 1 [file Data_Sheet_1.docx]

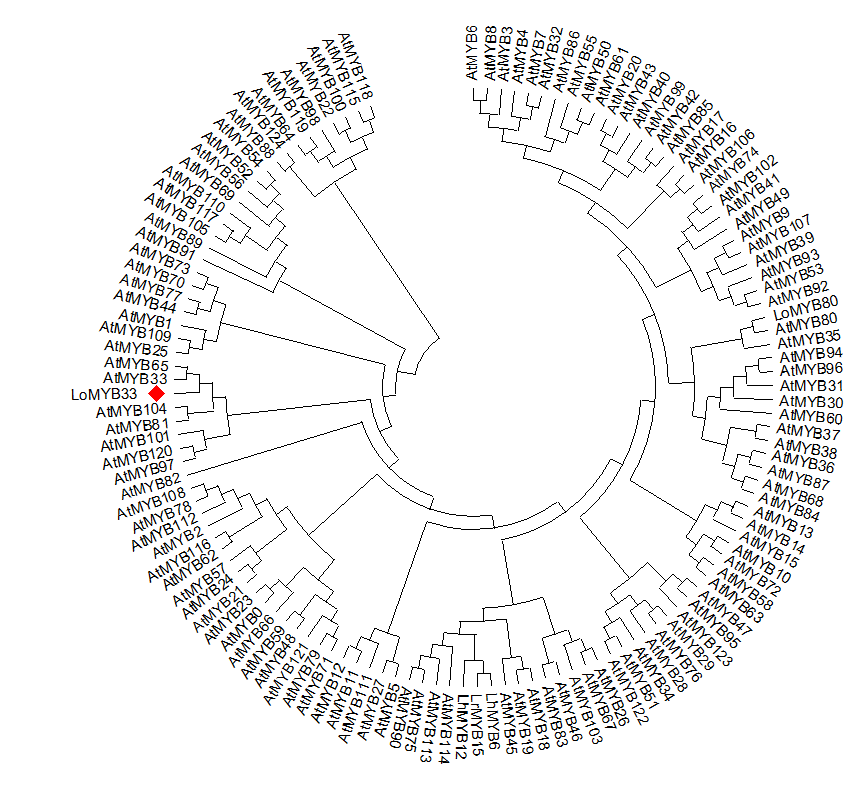


**Supplementary FIGURE 1.** The phylogenetic tree of LoMYB33 protein and Arabidopsis MYB transcription factor family from the TAIR database and 4 other MYB genes from lily, namely LrMYB15, LhMYB6, LhMYB12 and LoMYB80. The red diamond indicates LoMYB33. The phylogenetic tree of the MYB family of Arabidopsis was constructed using the neighbor-joining method of MEGA7 (Kumar et al., 2016). LrMYB15 (BAU29929), LhMYB6 (MK191027), LhMYB12 (MK182390), LoMYB80 (KF857220).


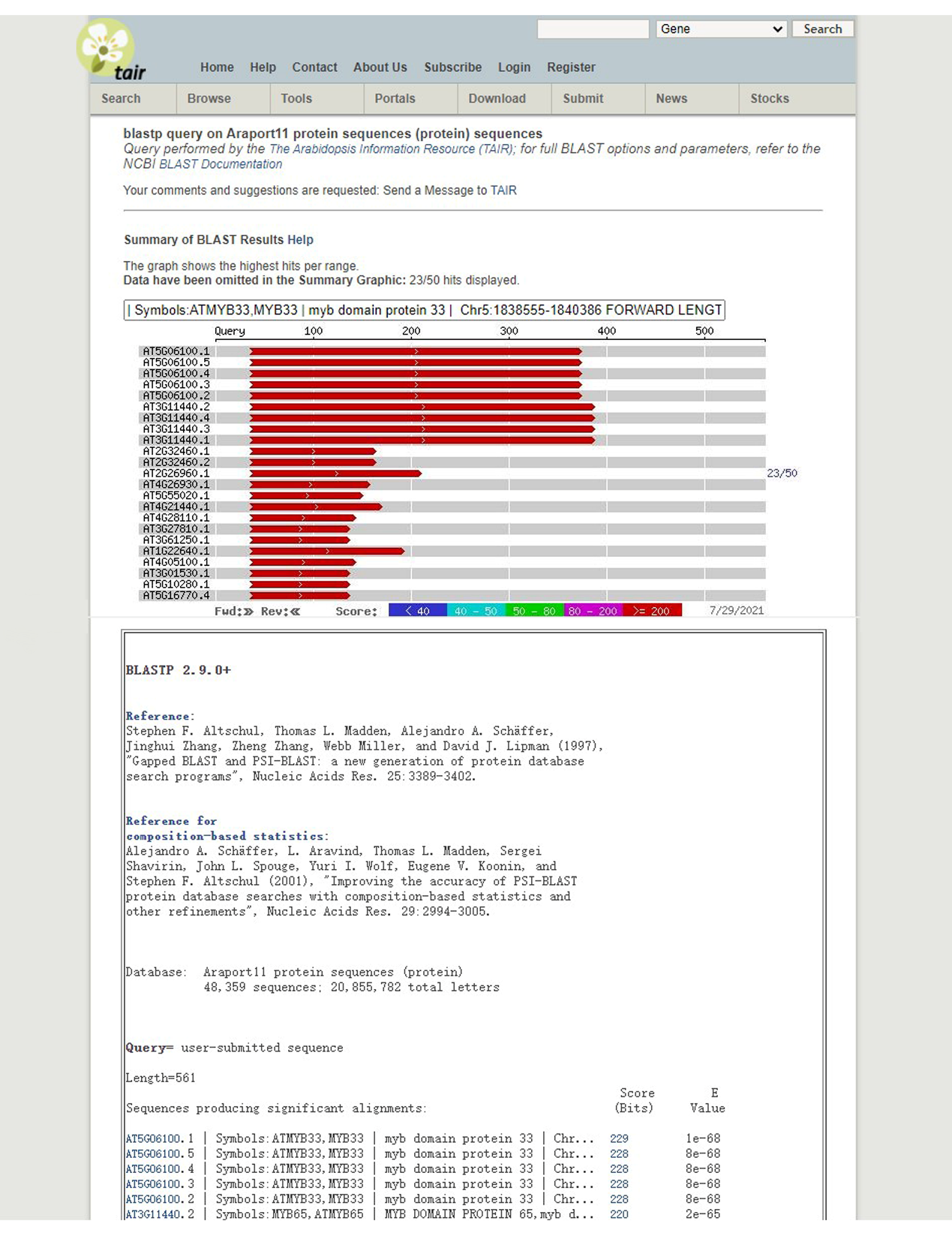


**Supplementary FIGURE 2.** The Blast results of LoMYB33 in Arabidopsis TAIR website (<https://www.arabidopsis.org/index.jsp>)


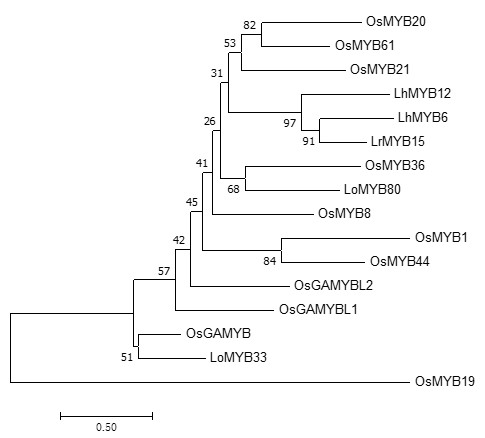


**Supplementary FIGURE 3.** Phylogenetic tree of LoMYB33 and some MYB proteins in rice and lily. OsGAMYB (CAA67000), OsGAMYBL1 (AB212075), OsGAMYBL2 (AAT76349), OsMYB1 (XP_015630688.1), OsMYB8 (XP_015638107.1), OsMYB19 (XP_015613057.1), OsMYB20 (XP_015610941.1), OsMYB21 (XP_015622999.1), OsMYB36 (XP_015648313.1), OsMYB44 (XP_015612590.1), OsMYB61 (XP_015627109.1), LrMYB15 (BAU29929), LhMYB6 (MK191027), LhMYB12 (MK182390), LoMYB80 (KF857220). The phylogenetic tree was constructed using the neighbor-joining method of MEGA7 (Kumar et al., 2016).


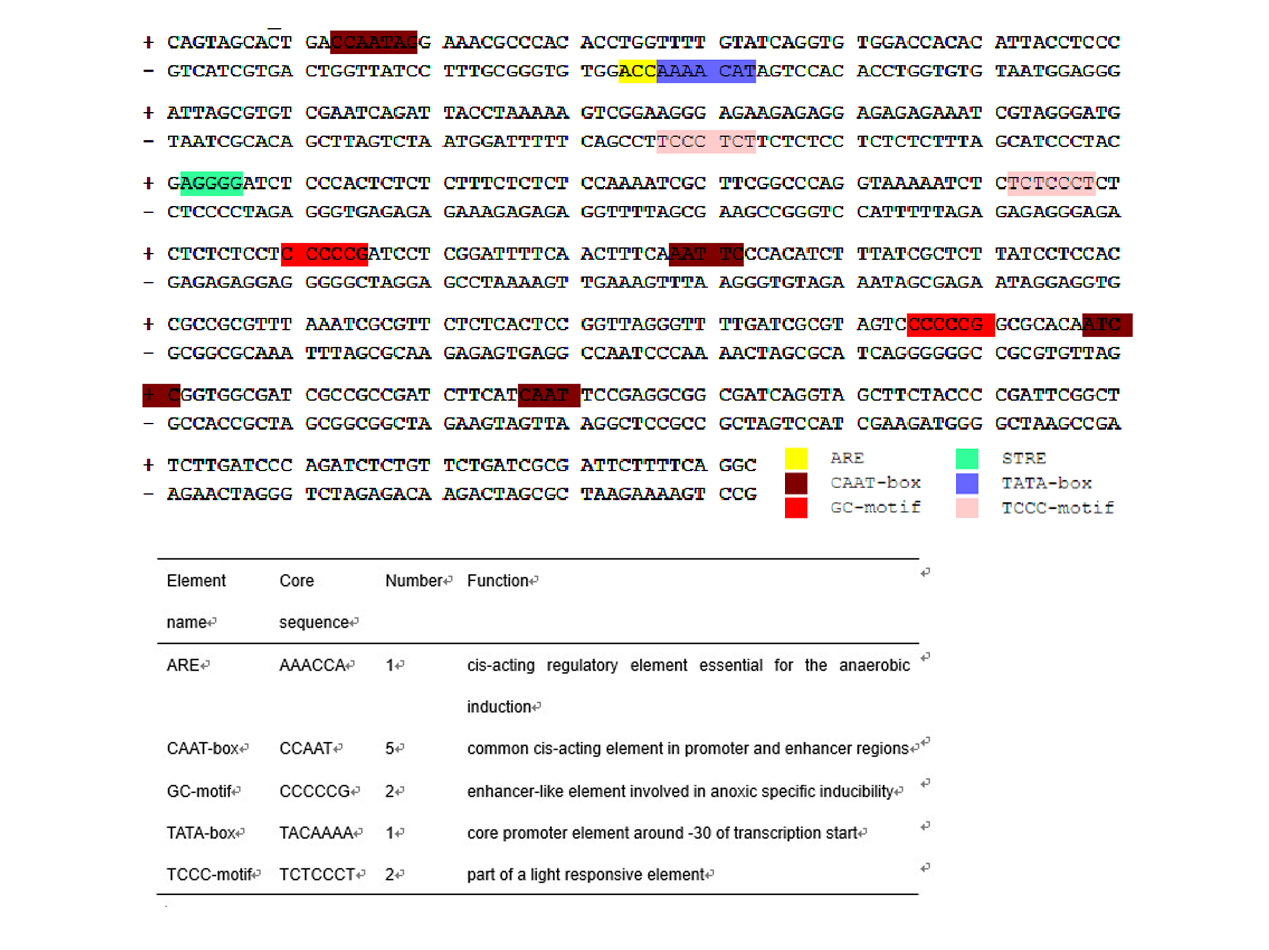


**Supplementary FIGURE 4.** Sequence analysis of *LoMYB33* promoter and the main cis-acting elements in the *LoMYB33* promoter. Yellow area indicates ARE, brown indicates CAAT-box, red indicates GC-motif, green indicates STRE, blue indicates TATA-box, and pink indicates TCCC-motif.


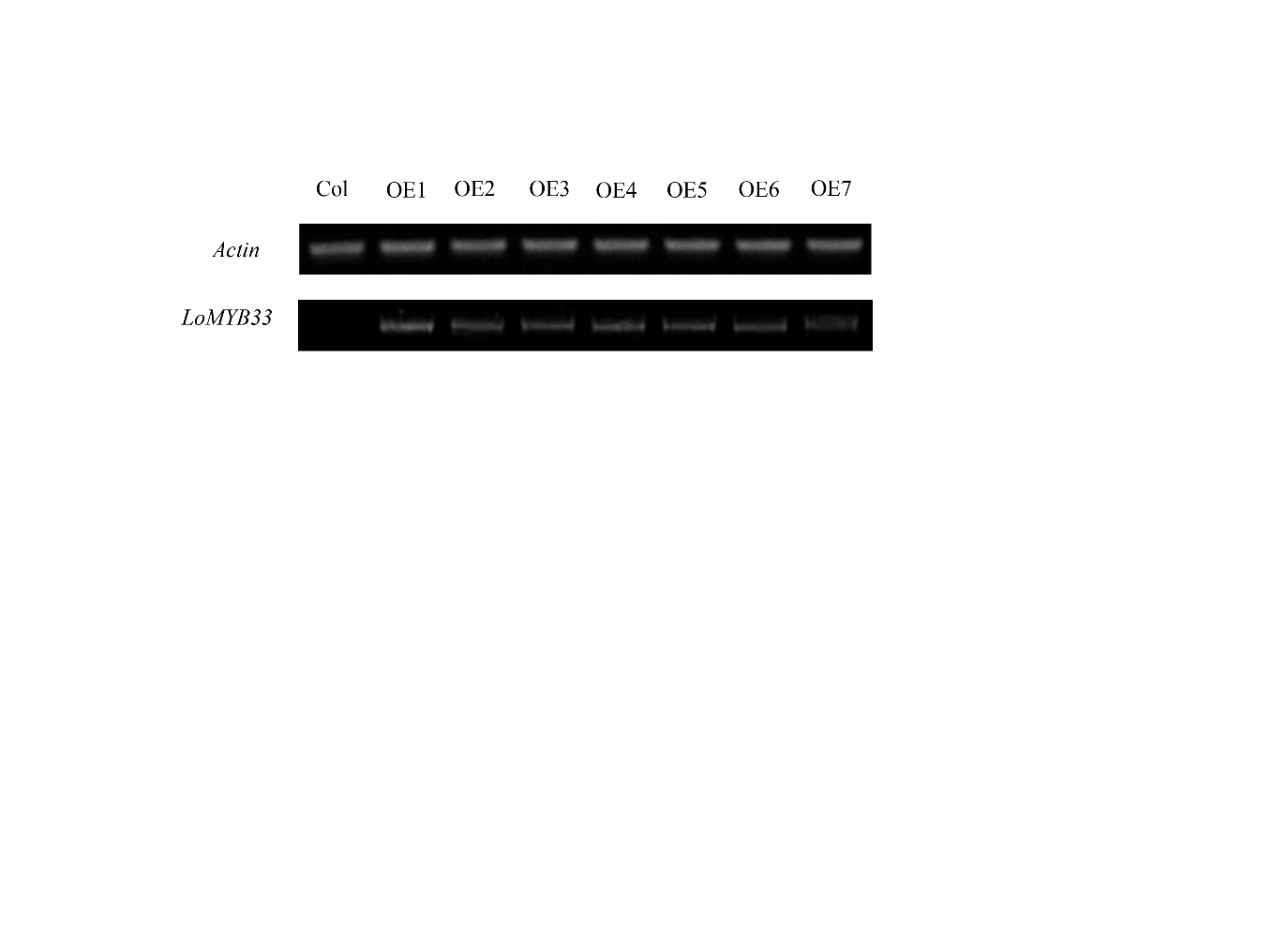


**Supplementary FIGURE 5.** Identification of *LoMYB33* transgenic lines by RT-PCR. PCR of the endogenous control and test gene was performed with 28 and 30 cycles, respectively. *AtActin2* was used as an endogenous control. Col, Wild-type plants, OE1-7, Overexpression lines of Arabidopsis*.*


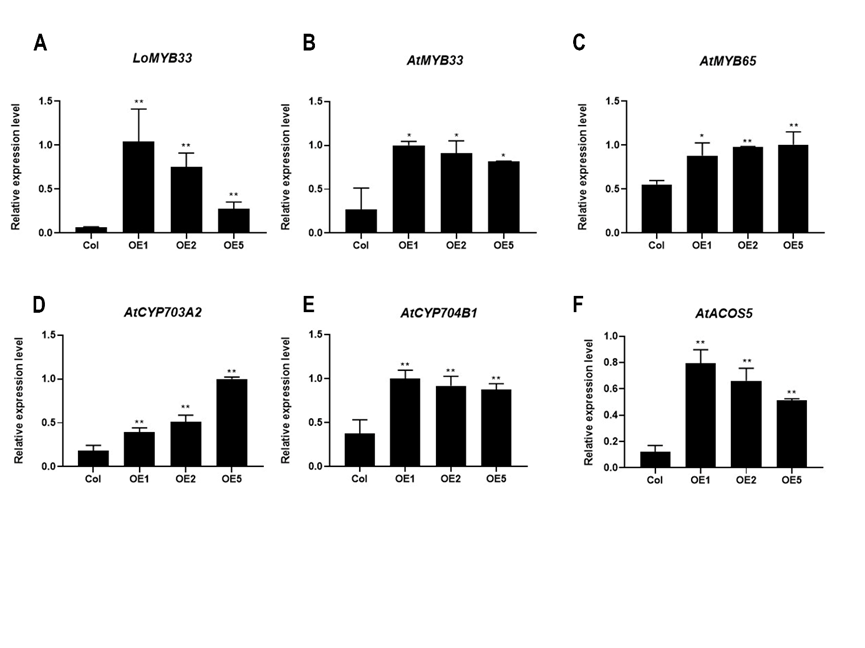


**Supplementary FIGURE 6.** Analysis of the expression of *LoMYB33*, *AtMYB33*, *AtMYB65*, *AtCYP703A2*, *AtCYP704B1*, and *AtACOS5* by RT-qPCR. (**A**) Expression of *LoMYB33* in OE lines was compared with that in wild-type plants. (**B**) The expression of *AtMYB33* in OE lines was compared with that in wild-type plants. (**C**) The expression of *AtMYB65* in OE lines was compared with that in wild-type plants. (**D**) The expression of *AtCYP703A2* in OE lines was compared with that in wild-type plants. (**E**) The expression of *AtCYP704B1* in OE lines was compared with that in wild-type plants. (**F**) The expression of *AtACOS5* in OE lines was compared with that in wild-type plants. Data are mean ± SD of three independent experiments (t-test, *P < 0.05, **P<0.01).


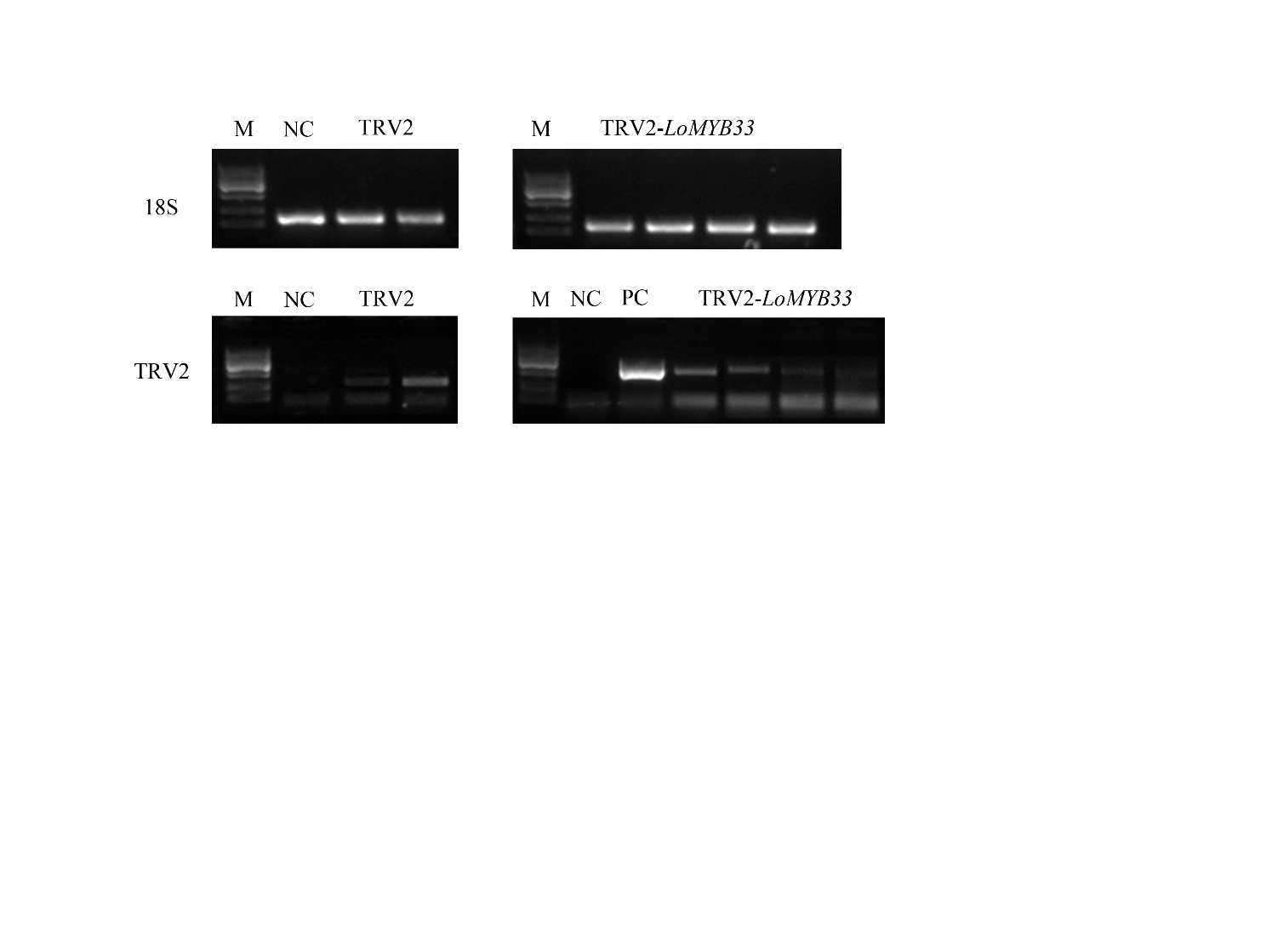


**Supplementary FIGURE 7.** Detection of TRV transcription in the VIGS experiment. DL2000 marker, NC, uninfected anthers; PC, pTRV2-*LoMYB33* *Agrobacterium* liquid; TRV2, TRV empty vector (pTRV1 + pTRV2) were used to infect anthers; TRV2-*LoMYB33*, pTRV2-*LoMYB33* (pTRV1 + pTRV2-*LoMYB33*) were the anthers infected by *Agrobacterium tumefaciens*.
