# Supplementary material for: A Novel R2R3-MYB Gene LoMYB33 From Lily Is Specifically Expressed in Anthers and Plays a Role in Pollen Development
Source: Front Plant Sci. 2021 Sep 23;12:730007. doi: 10.3389/fpls.2021.730007 (PMC8495421; doi:10.3389/fpls.2021.730007)
Supplement: Supplementary file 3 [file Table_2.DOCX]

>LoMYB33

MTLIKKESGKTPMPEERTDSPPSGGEPAAAGGDGLKKGPWTSMEDAILMEYVKKHGDGNWNNVQKFSGLSRCGKSCRLRWANHLRPNLKKGTITPEEERKIIQMHSQMGNKWARMAALLPGRTDNEIKNFWNTRMKRCERTGVPLYPDDVQFDPSNHNLRSQDVTNFGSIDKQYNEVMQGSTYGIIPEIVFENYKTDSGIPSYQSCFPNISGSTQGLGSRSPGLIDPSGNHLRRLRETETFLPGFQGSLHGFNGSSHYGGLPTCQQLPTWPAENVNQGFSLTGPFDPESSIMNPTPLGIVDPGSHALNGNFPSSRPLSGSAQLELPSFQNTDMEISNWIVCPSSPLPDPVDNFIHSPSAASLSPRNNGLLESLLPQVLSGSKNQQSLPELTPSNMLENSVLNHGEANWGDHEDPITPLGLTSDSVFSETPPFGGSPSDEFLFSQATSGKYFHAKNSKSMSKKTINTSFAGSEPMLAGSEHSSTPSFDELGSEWYNESYGGAKGRYMLNDVIASLLAGEMCSEDKQLPSGTAAASLPTVQGLGSECFPSSNMVPSNERPGAA

>PdGAMYB-like

MANSRLLSVCVNRRIDGELGFQESNVLKMNRTTDDSDNAMVPMDQVDSPSNEGSSGGLLTGGGQVLKKGPWTSAEDAILVDYVKKHGEGNWNAVQKHSGLSRCGKSCRLRWANHLRPNLKKGAFTPEEEKLIIELHARMGNKWARMAAHLPGRTDNEIKNYWNTRIKRRQRAGLPLYTSNVPFQASSENQQSQTATAYGNGEKRPNELLQGNSFDFPDAIFDSFNANHGALSYAPPFSDISVSSMLSQGFGSQNYGFMNPMGNHVKQFRESETSLPGFHGSVTTGLPPLEQFSNEPSEKIQRSFGLGYPYDPDPSSKDLAPFGGAIPGSHALSNGNFSASRPLGGTVKMELPSLQYPECDLSSWLACSSPPPEAIDNYIQSPPATASVQSECVSPRNSGLLDALLHEAQARKTQSSGKRSSSSVVAPGGMVESSGLNFCEAAWKEYNDPISPLGRPAASVFNECTPPVSGGSLDELQPSKEPSGSDIMLAAAEHVSTPNVGERDISPCSDFLRPDALLGGSDWLENSQTAKEHSTLNDAIATLLGEDLCSEYKEVPAGTSSTLSQGLGLDSCPWNNMPRACQMS

>EgGAMYB

MNRTTNDSDNIMVSKDQVDSPSNEGSSGGSLTGGGQVLKKGPWTSAEDAILVDYVKKHGEGNWNAVQKHSGLSRCGKSCRLRWANHLRPNLKKGAFTPEEEQLIIELHARMGNKWARMAAQLPGRTDNEIKNYWNTRIKRLQRAGLPLYPPNLSYQASSENQQSQTATEYGHGEKRPNELLQGNTSDLPDVMFESLNAHQGALSYAPPFPDISVSSMLTQGFGSQNYGFMNPMGNRMKRFRESETLLPVFDGSVSSALPTFQQFSDEPSEKIRRSFGLGYPYDPDPSSEIPTPFGGAVPGSHALSNGNFSASRPLGGTVKMELPSLQYSETDLSSWFACPSPPPEAIDSYIQSPPATASVQSECVSPRNSGLLDALLHEAQARKTQSSEKNSSSSVVAPGGMAESSGLPLCEAEWEEYNDPISPLGRPAASVFNECTPPVSGVSLDELQPSKEPSVPAGSDIMLEHVSASNMEGRDISACPDFLRPDALLGGSDWLENSQTAKEYSTLNDAIATLLGEDLCSEFKDVPAGASSTLSQGLGLDSCPWNNMPRACQMS

>MaGAMYB-like

MNGVPNESDRRMVPKDEIDSTSIEEDSSGGSLNGGNQVLKKGPWTSAEDAILIDYVKKHGEGNWNAVQKHTGLSRCGKSC

RLRWANHLRPNLKKGAFTPEEEQLIIELHAKMGNKWARMAAHLPGRTDNEIKNYWNTRIKRRQRAGLPLYPPNINFQVSD

ENQQSKNVNEYSYSDKQPNELMQGSNFDIPDITFENFNANYGSLSYAPPFPDIAASNMLNQRIGSLHYPFTNPMSCVKHL

RDSENLIPDLHGTDSDGLSTYEHFLFKPSGKIKQTFGLGYPYDPDPNGESLTPLESAVPGSHALLNGTFSASSPIDGTVK

LELPSLQYTETDGNSWLACSSTPLEAADTYIESPPTTVSLQSECISPRNSGLLEALLHEAQTLGSAKKKPSDKSSSSTIT

PGNVAECSGVSISEKLEEYNDPISPLGRSASSVFNEYTPPISGSSLDEFPHSMAPSTSDNILVTAKHVSSPNVVDKGISP

CRPDALLGSVWLDDGSQSAKDQSVFNDAIAILLGQDLCNEYKHLPSEPPSMLVQGFGLDSDPWNNMPSACQMP

>NnGAMYB

MGRSTNESEESMVLKVQTDSPSIDEGSCGGSMNGGVVLKKGPWTSAEDAILVDYVKKHGEGNWNAVQKHSGLFRCGKSCRLRWANHLRPNLKKGAFSQEEEKLIIELHAKMGNKWARMAAHLPGRTDNEIKNYWNTRIKRRQRAGLPLYPPELCLQALNESQQSQNSGEVNSGEKRHHDLLQGNSYEIPDVIFDNFKANQGALSFPSALPDISMSSMLTQGPGSSPNYSFMPPMMHRPKRLRESEALFPSYNGSITNGFPEFEQFQGDNCQKIHRPCGMSFPYDPDPSSKNPQHFGGHSLLNGNFSASKPTPGSMKLELPSLQYQETDLGGWGTSTLLPPSLESVDTFIQSPPPTGTVQSDCLSPRNSGLLEALLHEAQNLSSGKNHSFEKSSNSSVVTPSDVVDSSSLNLCDTEWEEYSDPISPLGRSAASVFSECTPISGSSLDETTPSDIIPGSRIKPETIENVSTPDGEKEISNRSNLSRPDALLGTDWFEQSTENGNQSVVNDAIATLLGEDFCNDYKQVPARSSTLPQGQGWSLGSCAWNNMPGVCHMSELP

>PtGAMYB

MSRTTSESGDGMISKDQTESPLGEEGSYGGSTNGVVLKKGPWTSAEDAILIEYVKKHGEGNWNSVQKHSGLFRCGKSCRLRWANHLRPNLKKGAFTQEEEQLIIELHAKMGNKWARMAAHLPGRTDNEIKNYWNTRIKRRQRAGLPLYPPEVSLQTLQGSQQCLDINGMDSGNKGQHDILQTHNYGIPDVMFDNLKTNRSILPYVPELPDISASSILMKGLCSSQYGSFMSPTMHRQKRLREATTLLSSFSGGMKNDFHLFDQFQDGDKAAQYFGFSFPFDPDSATKNPEIFGENQGSHTLANGNFSASKPTTEAVKLELPSLQYAEIDLGGWGESCSPSPLIESVDTFIQSPPTGTVESNFPSPRNSGLLDALLYEARTLSSAKNQSSDKSSNSSTITPGDNADCSALNFSETEWEDYGDPISPLGYPATSLFSECTPISAGGSSLDESPPAETLTGCNMKSEPVDHTWTADREKESYTQLDLTRPDALLDSDWLEHDSGYGKDQVIMTEAIATLLGDDLSSEYKQMAAGASTDHGWGLGSCSWNNMPAVCQMSELP

>VvGAMYB-like

MSHLTNESEDGMLSKDQTGSPLIDEGNSSGSAGGGIVLKKGPWTSSEDAILVEYVKKHGEGNWNAVQKHSGLFRCGKSCRLRWANHLRPNLKKGAFTAEEERLIIELHAKMGNKWARMAAHLPGRTDNEIKNYWNTRIKRRQRAGLPLYPPEVCFQALQESQQIQNTGGINGGDKVHHDLLQTNSYEIPDVILDGFKANQDAFPYVPDFPNLSTSSMLMKGFGSSQYCGFMAPTMHRQKRVRESPALFPGYNERNGIPLFDQIQDDTGDKIAQSFGLPYPFDPDPTNKDPLPFAGMQGSHALLNGNFSASKPISGPVKLELPSLQYLETDLGGWGTSSSPPPLLESVDAFIQSPPSGTVQLECLSPRNSGLLDALLHEAKTISSGKNHLSDKSANSSTVTPGDLADSCLNMCETEWEDYGDPISPLGHSTTSIFSECTPISASGSSLDEHPPSETFPGCNVKAEPVDQVWTPDGEKETSTQLDFSRPDVLLGSNWIDQSTGGPKDQAMMNDAVASLFSDDFGNNYKNMASGTPTSNQGWGLGCCAWNNLPPVCQMSELP

>AoGAMYB-like

MSDISNESDMKMLPRDHVGSPSIDEGSSGGSLAAGGGSGLKKGPWTSAEDAILVEYVKKHGEGNWNAVQKHSGLSRCGKSCRLRWANHLRPNLKKGAFTPDEEKTIIELHARMGNKWARMAAHLPGRTDNEIKNYWNTRIKRRQRAGLPVYPSDFSFPASDENQQGPTVGDFSSISKIQNESPQGNCYNITDIIFDDLKASYTPRFLPDISLSNVLSQGFGSLSYGFTTQPMDCKPFRGNETLFSFPASFENFSDDPSQKIDLTLGTSYPYDPDPIIKDVAPYEGAINGSHALMNGNYSTSKSLSGTVKSELPSLQFTETDPSFWFAYDSSPPSEAVDTYIQSASAVSMQSECSSPRNNGLLEDVLHESHAMHSGKKVPYEKCSNSSVMTPCDVMDSSANNFCEDTAFEHCDPISPLSCSAASVFNEYTPPISSSSLDELPPPRGPSGSDIILPSAEDVSTPNPLGGKIKPKPEFLRPDAILGSAWLLESSQMAKDHTDMNNAIATLLGDECCSEHKPLTTETSVSFTGALGLDSYPWNNMPRVCQISEHP

>AtMYB33

MSYTSTDSDHNESPAADDNGSDCRSRWDGHALKKGPWSSAEDDILIDYVNKHGEGNWNAVQKHTSLFRCGKSCRLRWANHLRPNLKKGAFSQEEEQLIVELHAKMGNRWARMAAHLPGRTDNEIKNYWNTRIKRRQRAGLPLYPPEMHVEALEWSQEYAKSRVMGEDRRHQDFLQLGSCESNVFFDTLNFTDMVPGTFDLADMTAYKNMGNCASSPRYENFMTPTIPSSKRLWESELLYPGCSSTIKQEFSSPEQFRNTSPQTISKTCSFSVPCDVEHPLYGNRHSPVMIPDSHTPTDGIVPYSKPLYGAVKLELPSFQYSETTFDQWKKSSSPPHSDLLDPFDTYIQSPPPPTGGEESDLYSNFDTGLLDMLLLEAKIRNNSTKNNLYRSCASTIPSADLGQVTVSQTKSEEFDNSLKSFLVHSEMSTQNADETPPRQREKKRKPLLDITRPDVLLASSWLDHGLGIVKETGSMSDALAVLLGDDIGNDYMNMSVGASSGVGSCSWSNMPPVCQMTELP

>AtMYB65

MSYTTATADSDDGMHSSIHNESPAPDSISNGCRSRGKRSVLKKGPWTSTEDGILIDYVKKHGEGNWNAVQKHTSLARCGKSCRLRWANHLRPNLKKGAFSQEEEQLIVEMHAKMGNKWAQMAEHLPGRTDNEIKNYWNTRIKRRQRAGLPLYPPEIYVDDLHWSEEYTKSNIIRVDRRRRHQDFLQLGNSKDNVLFDDLNFAASLLPAASDLSDLVACNMLGTGASSSRYESYMPPILPSPKQIWESGSRFPMCSSNIKHEFQSPEHFQNTAVQKNPRSCSISPCDVDHHPYENQHSSHMMMVPDSHTVTYGMHPTSKPLFGAVKLELPSFQYSETSAFDQWKTTPSPPHSDLLDSVDAYIQSPPPSQVEESDCFSSCDTGLLDMLLHEAKIKTSAKHSLLMSSPQKSFSSTTCTTNVTQNVPRGSENLIKSGEYEDSQKYLGRSEITSPSQLSAGGFSSAFAGNVVKTEELDQVWEPKRVDITRPDVLLASSWLDQGCYGIVSDTSSMSDALALLGGDDIGNSYVTVGSSSGQAPRGVGSYGWTNMPPVWSL

>OsGAMYB

MYRVKSESDCDMIHQEQMDSPVADDGSSGGSPHRGGGPPLKKGPWTSAEDAILVDYVKKHGEGNWNAVQKNTGLFRCGKSCRLRWANHLRPNLKKGAFTAEEERLIIQLHSKMGNKWARMAAHLPGRTDNEIKNYWNTRIKRCQRAGLPIYPTSVCNQSSNEDQQCSSDFDCGENLSNDLLNANGLYLPDFTCDNFIANSEALPYAPHLSAVSISNLLGQSFASKSCSFMDQVNQTGMLKQSDGVLPGLSDTINGVISSVDQFSNDSEKLKQAVGFDYLHEANSTSKIIAPFGGALNGSHAFLNGNFSASRPTSGPLKMELPSLQDTESDPNSWLKYTVAPALQPTELVDPYLQSPAATPSVKSECASPRNSGLLEELIHEAQTLRSGKNQQTSVISSSSSVGTPCNTTVLSPEFDMCQEYWEEQHPGPFLNDCAPFSGNSFTESTPPVSAASPDIFQLSKVSPAQSTSMGSGEQVMGPKYEPGDTSPHPENFRPDALFSGNTADPSVFNNAIAMLLGNDLSIDCRPVLGDGIMFNSSSWSNMPHACEMSEFK

>RcMYB33

MSRTTTDSEDGLLSKDQIESPLMDESNGGIGNGGIVLKKGPWTSAEDAILVEYVKKHGEGNWNAVQKHSGLFRCGKSCRLRWANHLRPNLKKGAFTPEEERLIVELHAKMGNKWARMAAHLPGRTDNEIKNYWNTRIKRRQRAGLPLYPPEVCLQALQESQQGQSSGALNGTDTAHHDSLQTNSYEIPDVVFDSLKGNSCVLPYVPELPDISASGMLMKGLGSSPYCGFMPPTMHRQKRPRESTALFPTSGGSYKDGFPQFDQFENDADRVARSYGLSFPHDPDPTTKSPLSFGVIQGSHSLSNGNSSASKPTCGAVKLELPSLQYPETDLGSWSTSPPPPLLESIDNFIQSPPPVGAFESDCASPRNSGLLDALLYEAKTLGSTKNHSTDKSSNSSSVTPGEVADSSTLNVCETEWEEYGDPISPLGHSATSLFSECTPISASGSSLEEVPPAETLTGSNVKPEPADHAWTPEEQKEASPPLDYTRPDALLGSDWLEPSTGFKDQSIMNDAIASLLGEDLATDYKHMASGTSTSNQGWGLGSCPWNNMPAVCQMTDLP

>TaGAMYB

MYRVKSESDCEMMHQEDQMDSPVGDDGSSGGSPHRGGGPPLKKGPWTSAEDAILVDYVKKHGEGNWNAVQKNTGLFRCGKSCRLRWANHLRPNLKKGAFTPEEERLIIQLHSKMGNKWARMAAHLPGRTDNEIKNYWNTRIKRCQRAGLPIYPASVCNQSSNEDQGSSDFNCGENLSSDLLNGNGLYLPDFTCDNFIANSEALSYAPQLSAVSISSLLGQSFASKNCGFMDQVNQAGMLKQSDHLLPGLSDTINGALSSVDQFPNDSEKLKKALGFDYLHEANSSSKTIAPFGGALTGSHAFLNGTFFTSRTINGPLKMELPSLQDTEFDPNSWLKYTVAPAMQPTELVDPYLQSPTATPSVKSECASPRNSGLLEELLHEAQGLRSGRNQQLSVRSSSSSVSTPCDTTVISPEDLCQEYWEERLNEYAAPFSGNSLTGSTAPVSAASPDVFQLSKLSPGSPSLGSGEQAMEPAYEPGAGDTSSHPENFRPDAFFSGNTTDSSVFNNAIAMLLGNDMNTDCKPVFGHGIVFDTSPWSNMPHACQMSEEFK

>ZmGAMYB

MYRVKSEGEGEGEGDCEMMLQEQMDSLVADDVSSGGGSPHRGVGTPLKKGPWTSAEDAILVDYVKKNGEGNWNAVQKNTGLFRCGKSCRLRWANHLRPNLKKGAFTPEEERLIIQLHAKMGNKWARMAGHLPGRTDNEIKNYWNTRIKRCQRASLPIYPASVCNQSTNEDQQLSGNFNGGENISNDLLSGNSLYLPDFTSDNFIANPEALSYAPQLSAVSISNLLGQSFASKSCSFMDQVDQAGMLKQSGCVLPALSDAIDSVLSSADHFSNDSEKLRQALGFDYLNEANASSKSIAPFGVALTGSHAFLNGNFSASRPTNGPLKMELPSLQDTESDPNSWLKYTVAPAMQPTELVDPYLQSPSATPSVKSECASPRNSGLLEELLHEAQALRSGKNQQSSVRSSSSSAGTPYETTTVVSPEFDMGQEYWEEQPSSFLSEYAHFSGNSFTESTPPVSAASPDIFQLSKISPAQSPSMGSGEQALEPKHESAASPRPENLRPDALFSGNTADPSIFNNAIAMLLGNGIDAEYKPVLGDGIVLDSSSWNNMQHAFQMAGFK

>HvGAMYB

MYRVKSESDCEMMHQEDQMDSPVGDDGSSGGGSPHRGGGPPLKKGPWTSAEDAILVDYVKKHGEGNWNAVQKNTGLFRCGKSCRLRWANHLRPNLKKGAFTPEEERLIIQLHSKMGNKWARMAAHLPGRTDNEIKNYWNTRIKRCQRAGLPIYPASVCNQSSNEDQQGSSDFNCGENLSSDLLNGNGLYLPDFTCDNFIANSEALSYAPQLSAVSISSLLGQSFASKNCGFMDQVNQAGMLKQSDPLLPGLSDTINGALSSVDQFSNDSEKLKQALGFDYLHEANSSSKIIAPFGGALTGSHAFLNGTFSTSRTINGPLKMELPSLQDTESDPNSWLKYTVAPAMQPTELVDPYMQSPTATPSVKSECASPRNSGLLEELLHEAQGLRSGKNKQLSVRSSSSSVSTPCDTTVVSPEFDLCQEYWEERLNEYAPFSGNSLTGSTAPMSAASPDVFQLSKISPAQSPSLGSGEQAMEPAYEPGAGDTSSHPENLRPDAFFSGNTADSSVFNNAIAMLLGNDMNTECKPVFGDGIMFDTSVWSNLPHACQMSEEFK

>GmGAMYB1

MRRMKKDIEDEVLPNDMSGAQLNDESYEGSAGIVLKKGPWTSTEDDILVDYVKKHGEGNWNAVQKHTGLFRCGKSCRLRWANHLRPNLKKGAFAAEEERLTAELHAKMGNKWARMAAHLPGRTDNEIKNYWNTRIKRRQRAGLPLYPPEVSLQALQESQHSQSTGGLNGGDKMHPDFLQKNSYEIHDAIFDSLKDNQGILPYVHELSDISVYGNKLKGLDSSQYCSFVPPTSPKRKRLKESTIPFIDSCAMKKKDLYPFDQIQDNNSDKIAQSFGMQAPLDPGLSSHSSMCYSHSLSNGNSSTSKPYEAMKLELPSLQYPELDLGSWGSSPPPSLLESVDDFLQSPTAISTLESDCSSPQNSGLLDALLYQAKTMSSSKNHCSDKSSNSSTATPGDRADSSILNVYETEWEDYADPVSPFGATSILNECPALRANANSLNGQLPVQTLTGNLPKLESVDQVWTPNNENLTLSLLNITRPDFLLASDWYDLGSGHCKNQTITTDAATAFLGDDLATDLKHMTAGISKTMFENPFQPL

>CsGAMYB1

MRHPKNEIEDNLPSQDQTLSPLLDEDSGGNASGIILKKGPWTSAEDEILIEYVKKHGEGNWNAVQKHSGLSRCGKSCRLRWANHLRPNLKKGAFTAEEEHLIIELHAKMGNKWARMAGHLPGRTDNEIKNYWNTRIKRRQRAGLPLYPPEVCLRTWQALQQTQDSGGSTVVDTDHHDLLRSNSYDIPDVTFHSLKPQSALSYMPELPDISSCMLKRGLDTSQYCNLVQPTFHRQKRFRDSASLFPGPDGSVKTPFHQFEDNSYSQAAQSFGTPFAHESNPTTKNAMSFGSFEGSHSLTNGNSSASQHSKETEKLELPSLQYPETDLTSWDTTIQPAMFESVDPFIQSTPTFVLAPDRTSPCHSGLLESLVYSKTMGPKNHPSDKNSNSCSVTPGDVTDSYNMAASKTEIDDYTEVISPFGHSTSSLFSECTPISATGSSYEDPTLTEAFSGSHVKSEPFDHAWTPDREKAAKSRVNFARPDALLASDWHDRSSGIVEDTTNVTDAISLLLGDDLAADYEHFPNGISTTHSAWGLDSCSWNNMPAVCHMSDLP

>SIGAMYB1

MSIKSETEERMTSKVDMDSPDEASGGDLGESVPLKKGPWTSAEDVILVDYVMTHGEGNWNAVQRHSGLARCGKSCRLRWANHLRPDLKKGAFTPEEEQRIVELHAKMGNKWARMAVELPGRTDNEIKNYWNTRIKRRQRAGLPIYPADISFMASQNKQNEELGAFSSADAQNPDVLGINNFEIPAVEFKKLELTHLLYPPQLADIPARSLLNDPVSNFLSQGHRAPYSSTYFLSTTYPAKRIRGSESVFSGSNGDLLNSLQYQNDGSLLAQAQAQPLDFSSYNHNLTYDDQRAISNIVPGGHAYLNGNSSSEPTWAMKLELPSLQNQTENWGSPHSALPSLDSVDILIQSPPAGHSESGSLSPSNSGLLDAVLHESQTMKASNDNSYQGNETSGNAVNNSCPDLKGCDIYGHPVSPLSQFSASVFSDYAPINESSLHEFPSMATMPGGEIKQEIGDLSPLDDEDNTSNQTIFSSPKTQHANNHLASKDPFGSCFFDDCDWDCKQIHAVTTSSGQANGHNSCSWDAISAMEATGRMRL*

>SIGAMYB2

MSMTSESDDRMTSQDGVDSPSAEEACGGGNTGGGLPLKKGPWTSAEDAILVEYVTKHGEGNWNAVQKHSGLARCGKSCRLRWANHLRPDLKKGAFTPEEERHIIELHAKMGNKWARMAAELPGRTDNEIKNYWNTRIKRRQRAGLPIYPSDICFQSITENKQNEELGTFSSADSQYPDFFPMNYEIPAVEFKRLEFNQHLCPPALLDIPTGGILDIPGRSLLAQGLNSAYYSRSFLSTTPPAKRIRGSESLFSGLNGDCSPSKNDVSFSTCHQHQDDGSLLAQSMGFSSSFNQNLTSDYHPSSLGVIPGSHALLNGHTSSSEPSWAKKLELPSLQSTIASWGLVTSPLPSLDSVDTLIQSPPTEHTESCNLSPRNSGLLDAVLHESQTMKASKSILHQENSGDVVDNSCPDLHMTEWGQHGDPISPLGHSAASVFSEYTPTSGSSSEEPQLVTMPACKVKQEKFDYGPYDGKDDASNLICPRPDFLLESNCFGHMQNTVRSIWY*
